# *Escherichia coli* adaptation and response to exposure to heavy atmospheric pollution

**DOI:** 10.1038/s41598-019-47427-7

**Published:** 2019-07-26

**Authors:** Tian Zhang, Xiao-Chen Shi, Yangyang Xia, Liqiang Mai, Pier-Luc Tremblay

**Affiliations:** 1 State Key Laboratory of Silicate Materials for Architectures, Wuhan University of Technology, Wuhan, P.R. China; 2 School of Chemistry, Chemical Engineering and Life Science, Wuhan University of Technology, Wuhan, P.R. China; 3 State Key Laboratory of Advanced Technology for Materials Synthesis and Processing, International School of Materials Science and Engineering, Wuhan University of Technology, Wuhan, P.R. China

**Keywords:** Bacterial genomics, Environmental microbiology, Bacterial genetics, Environmental impact

## Abstract

90% of the world population is exposed to heavy atmospheric pollution. This is a major public health issue causing 7 million death each year. Air pollution comprises an array of pollutants such as particulate matters, ozone and carbon monoxide imposing a multifactorial stress on living cells. Here, *Escherichia coli* was used as model cell and adapted for 390 generations to atmospheric pollution to assess its long-term effects at the genetic, transcriptomic and physiological levels. Over this period, *E*. *coli* evolved to grow faster and acquired an adaptive mutation in *rpoB*, which encodes the RNA polymerase β subunit. Transcriptomic and biochemical characterization showed alteration of the cell membrane composition resulting in lesser permeability after the adaptation process. A second significant change in the cell wall structure of the adapted strain was the greater accumulation of the exopolysaccharides colanic acid and cellulose in the extracellular fraction. Results also indicated that amino acids homeostasis was involved in *E*. *coli* response to atmospheric pollutants. This study demonstrates that adaptive mutation with transformative physiological impact can be fixed in genome after exposure to atmospheric pollution and also provides a comprehensive portrait of the cellular response mechanisms involved.

## Introduction

9 out of 10 persons are breathing heavily polluted air worldwide^1^. Morbidity and mortality have been shown to be increased after exposure to fine particulate matter (PM) found in air pollution^2,3^. According to the World Health organization, heavy atmospheric pollution causes 7 million death each year via a wide range of diseases including heart disease, stroke, lung cancer, respiratory infections and chronic obstructive pulmonary diseases^4,5^. Fine PM, which is considered to be the principal factor responsible for the relation between atmospheric pollution and morbidity, affects health negatively via multiple mechanisms such as genotoxicity, inflammation, cell death, procoagulant effects and reactive oxygen species (ROS)^6,7^.

Air pollution composition varies significantly depending on the geographical area, the season and the source of pollutants^8^. Generally, it contains PM with a diameter between 2.5 to 10 μM (PM_10_), PM below 2.5 μM (PM_2.5_), ultrafine PM (PM_0.1_), ozone, carbon monoxide, nitrogen oxides, sulfur oxides, metals, organic compounds and biological matter^9^. PM_2.5_ and PM_0.1_ are the most toxic PM because they are respirable and can penetrate deeper in the body^10^. The surface of PM is coated with metals and polycyclic aromatic hydrocarbons (PAHs), both of which can cause the formation of ROS and reactive nitrogen species (RNS)^11^. ROS triggers inflammation, damages cell membrane and DNA, alters enzyme activity as well as cell signaling^4^. Additionally, ROS generates supplementary reactive species when in contact with living cells. For instance, ROS degrades lipids in the cell membrane via peroxidation, which results in the formation of aldehydes and reactive electrophile species (RES)^4,12,13^. Metals on PM also substitute for polyvalent cations interfering with cell functions such as enzyme activity, gene expression, maintenance of protein conformation and membrane potential. Ozone comprised in atmospheric pollution is another ROS generator^9^.

Beside RES generated via the contact of ROS with cells, atmospheric pollution already contains exogenous RES such as (methyl)glyoxals, quinones and quinones-like molecules^14–16^. PAHs present at the surface of PM will also be (photo)chemically transformed into quinones^17^. RESs damage DNA as well as proteins and alter the intracellular redox balance by interfering with cofactors such as glutathione and NADH^18,19^. RESs are thought to be involved in several illnesses including diabetes and neurodegenerative diseases^20^.

Adaptive laboratory evolution (ALE) is an effective approach to gain insight on how living cells adapt and become fitter when facing a selective pressure such as a specific substrate, growth medium, electron acceptor, environmental stress or genetic perturbation^21–28^. ALE mainly consists in cultivating an organism under specific conditions over multiple generations^29^. During this process, mutations occur and beneficial ones are fixed in the genome^21^. ALE in combination with genome sequencing and transcriptomic has been used to study the impact of stresses such as alcohol, temperature, UV light, osmotic pressure and antibiotic on prokaryotic as well eukaryotic cells^30–34^.

Here, ALE was employed to elucidate the adaption process of living cells to heavily polluted atmosphere, which contains many potential sources of stress. The model bacterium *Escherichia coli* was exposed to polluted air from urban center or from diesel exhaust. *E*. *coli* was selected for this project because of the considerable body of scientific knowledge on its metabolism, physiology and, more specifically, on its response to stresses, which provides a good starting point to further understand cellular mechanisms involved in response to atmospheric pollution. Genome sequencing, transcriptomic and biochemistry were employed to pinpoint metabolic and physiological responses in the wild type (wt) as well as in a strain (T56-1) adapted by ALE to exposure to atmospheric pollution. A single mutation in *rpoB*, the gene coding for the RNA polymerases was detected in T56-1. This genetic alteration had a transformative impact on the transcriptome with 260 genes differentially-expressed. A series of adaptive mechanisms was discovered including change in the cell membrane composition, increase of exopolysaccharides, adjustment in the amino acids metabolism and augmentation of the enzymatic capacity for the degradation of toxic pollutants.

## Results

### Adaptation of *Escherichia coli* to heavy atmospheric pollution

**Figure 1:**
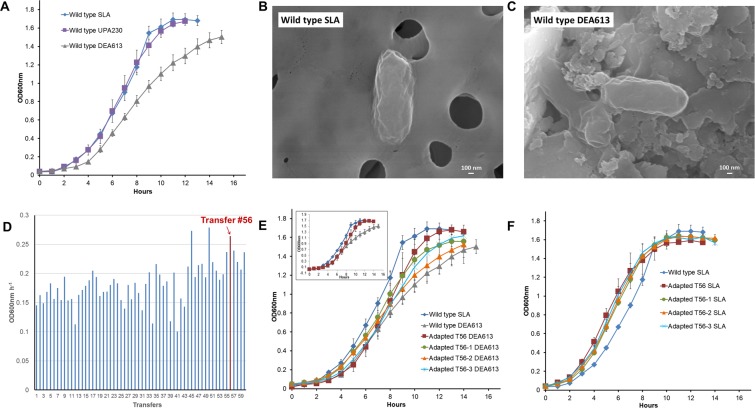
Growth and adaptation of *E*. *coli* BW25113 under heavy atmospheric pollution. (**A**) Wild type grew under SLA, UPA230 and DEA613. SEM images of wild type grew under (**B**) SLA or (**C**) DEA613. (**D**) Growth rates of different transfers under DEA613. Growth of DEA613-adapted T56 culture and purified clones under (**E**) DEA or (**F**) SLA. Each growth curves are the mean of three replicates.

The impact of atmospheric pollution on the genome, transcriptome and physiology of living cells was studied with the model bacterium *Escherichia coli* BW25113 (wt). Bacterial cultures were grown under standard laboratory atmosphere (SLA), urban polluted atmosphere with a PM_2.5_ concentration of 230 μg m^−3^ (UPA230) considered as very unhealthy or under diesel exhaust atmosphere with a PM_2.5_ concentration of 613 μg m^−3^ (DEA613), which is outside the air quality index range (AQI) (Table 1). Diesel exhaust was employed to simulate days with polluted atmosphere above the AQI, which are occasional in major cities. For instance, 10 days in Beijing and 27 days in Shanghai have been above the AQI from January 2017 to September 2018^35^. Exposure of *E*. *coli* wt to UPA230 had no discernible effect on growth in minimal medium with glucose as the substrate (Fig. 1A and Table 2). When the same strain was grown under DEA613, doubling time was 1.6 time slower compared to SLA-grown wt cultures. PM interacting with the surface of *E*. *coli* cells can be easily observed by SEM with the DEA613-grown cultures, but not with the SLA-grown cultures (Fig. 1B,C).

**Table 1:** Composition of standard laboratory atmosphere, urban polluted atmosphere and diesel exhaust atmosphere.

Atmosphere	PM_2.5_^a^	PM_10_^a^	SO_2_^a^	NO_2_^a^	O_3_^a^	CO^b^
Standard laboratory air (SLA)	17	19	N.D.^c^	23	0	N.D.
Urban polluted atmosphere (UPA230)	230	107	10	70	36	1.5
Diesel exhaust atmosphere (DEA613)	613	689	N.D.	8650	3930	294

^a^μg m^−3^, ^b^mg m^−3^, ^c^Not detected.

**Table 2:** Doubling time, lag time and maximal OD 600 nm for *E*. *coli* wild type and DEA613-adapted strain^a^.

Strain	Doubling time (min.)	Lag time (hour)	Maximal OD 600 nm
**Standard laboratory (SLA)**			
Wild type	62.3 ± 2.7	2.9 ± 0.1	1.7 ± 0.1
T56 culture	65.1 ± 4.1	1.6 ± 0.2	1.6 ± 0.0
T56 clone #1	65.3 ± 2.7	2.0 ± 0.1	1.6 ± 0.0
T56 clone #2	66.5 ± 4.4	2.0 ± 0.2	1.6 ± 0.0
T56 clone #3	66.8 ± 3.4	1.9 ± 0.2	1.7 ± 0.0
**Diesel exhaust atmosphere (DEA613)**			
Wild type	97.1 ± 3.8	2.8 ± 0.1	1.5 ± 0.1
T56 culture	78.7 ± 5.1	3.5 ± 0.3	1.7 ± 0.0
T56 clone #1	70.0 ± 6.5	2.5 ± 0.2	1.5 ± 0.0
T56 clone #2	85.2 ± 5.7	2.6 ± 0.3	1.5 ± 0.2
T56 clone #3	83.8 ± 4.4	3.5 ± 0.2	1.6 ± 0.1
**Urban polluted atmosphere (UPA230)**			
Wild type	66.6 ± 5.3	3.2 ± 0.3	1.7 ± 0.0

^a^Each value is the mean and standard deviation of three replicates.

Because of the observed growth defect, longer term cultivation of *E*. *coli* wt was performed under DEA613 to assess if the bacterium could become fitter under heavy atmospheric pollution. ALE was employed and *E*. *coli* wt batch cultures were transferred to fresh M9-glucose medium each time when they reached the beginning of the exponential growth phase. The strain employed for all the following experiments was the 56^th^ transfer (ca. 390 generations) as no further progress in growth rate was observed after that (Fig. 1D). The bacterial culture T56 grew 1.3 times faster than *E*. *coli* wt under DEA613 but not as fast as *E*. *coli* wt under SLA (Fig. 1E, Table 2 and Fig. S1). After streaking the T56 culture, three isolated clones still grew faster than *E*. *coli* wt under DEA613 with doubling times of 70.0 ± 6.5, 85.2 ± 5.7 and 83.8 ± 4.4 min, respectively. To ensure that *E*. *coli* became fitter to grow under the multifactorial stress associated with exposure to DEA613 and not only adapted to grow faster on M9-glucose medium, the T56 culture and the isolated clones T56-1, T56-2 and T56-3 were also cultivated under SLA (Fig. 1F and Table 2). There was no significant difference in doubling time among the adapted strains and the wild type. However, the adapted strains had a shorter lag phase.

The genome of adapted strains T56-1 and T56-3 was sequenced by whole-genome sequencing to investigate the cause of the fitness improvement under DEA613. Only one mutation was detected in both clones, which was a single nucleotide polymorphism (SNP) (CGT → CTT) resulting in the substitution of an arginine by a leucine (R12L) at position 12 of RpoB, the RNA polymerases β subunit (Fig. S2). Although progression could be observed in growth rate throughout the ALE experiment, clones from transfers prior to T56 were not sequenced since only one mutation was observed at this final stage (Fig. 1F). The presence of RpoB R12L in T56-1 and T56-3 was confirmed by Sanger sequencing.

RpoB comprises 1342 amino acids and is one of the five subunits forming the RNA polymerase responsible for RNA synthesis^36^. This subunit is involved in RNA synthesis and interacts with DNA as well as with nascent RNA in a nonsequence-specific manner^37^. Adaptive mutations in RpoB have been observed for several ALE experiments where *E*. *coli* population was evolved at higher temperature or for rapid growth in M9-glucose medium^24,38,39^. However, RpoB mutation in those studies were not located at the N-terminal end of the protein like the one found here.

### Differential gene expression in the adapted strain

Mutations in RpoB often have an impact on the expression of hundreds to thousands of genes causing pleiotropic effects on the cell physiology^40,41^. RNA sequencing experiments were conducted to establish how the adapted strain became fitter under DEA. Comparison of transcript abundance between T56-1 and the wt strain grown under DEA613 indicated that 260 genes were differentially expressed when the cut-offs were log_2_ fold change ≤1.5 or ≥1.5 and q-value ≤ 0.05. Additionally, to further understand how *E*. *coli* cells responded to atmospheric pollution, RNA was also sequenced with wt strain cultures grown under SLA and under UPA230. When transcript abundance was compared between SLA-grown wt versus UPA230-grown wt or versus DEA613-grown wt, 77 and 14 genes were differentially expressed, respectively. Out of those genes, only four were differentially-expressed in both wt grown under DEA613 or UPA230 compared to SLA (Table S1).

### Adaptation of the cell membrane

**Figure 2:**
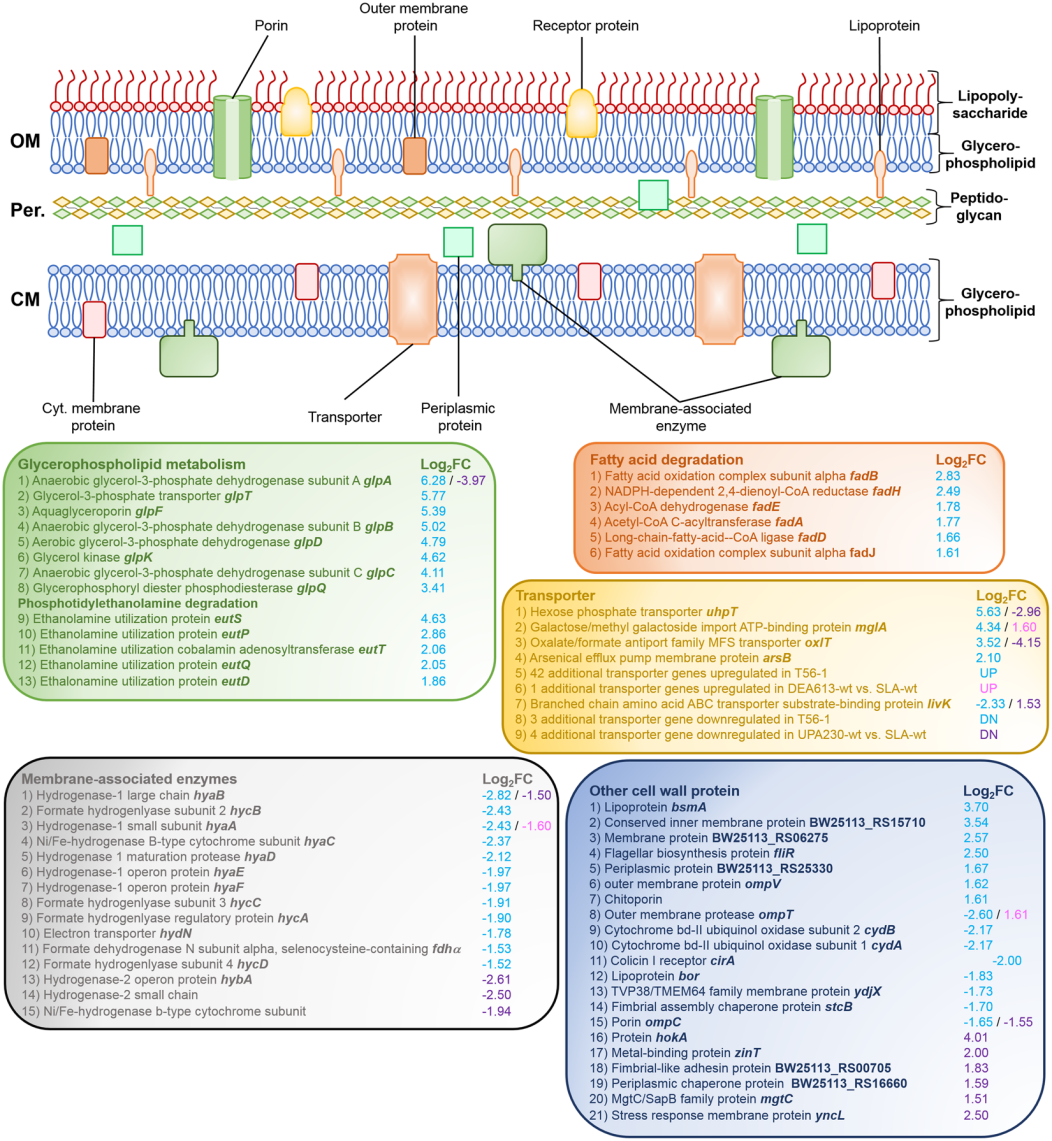
Differentially-expressed genes coding for proteins involved in the cell wall structure. Log_2_ fold change (Log_2_FC) values represent adapted T56-1 versus wt both grown under DEA613 (blue), UPA230-grown wt versus SLA-grown wt (purple), and DEA613-grown wt versus SLA-grown wt (pink), respectively. OM: outer membrane, Per.: periplasm, CM: cytoplasmic membrane.

**Figure 3:**
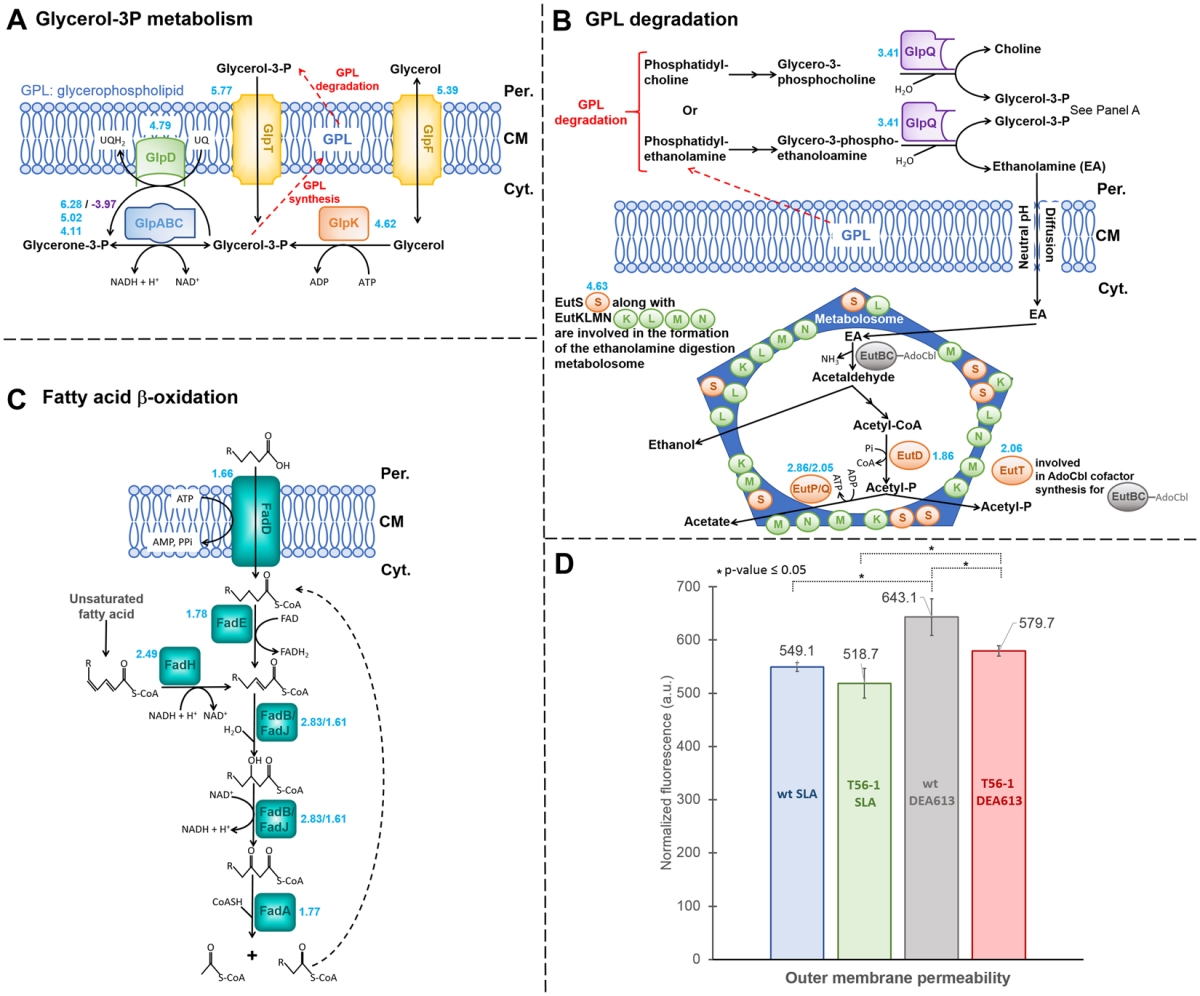
Cell membrane changes in the DEA613-adapted strain T56-1. (**A**) Differential expression of genes involved in the glycerol-3-phosphate (glycerol-3P) metabolism^42^, (**B**) in glycerophospholipid degradation, and (**C**) in fatty acid β-oxidation. Log_2_FC values represent adapted T56-1 versus wt both grown under DEA613 (blue), and UPA230-grown wt versus SLA-grown wt (purple), respectively. (**D**) Outer membrane permeability. Higher fluorescence caused by NPN insertion in the membrane corresponds to higher OM permeability. Fluorescence values were normalized with OD 600 nm and results are the mean and standard deviation of three replicates. Cyt.: cytoplasm.

When comparing T56-1 with wt under DEA613, the most upregulated gene was *glpA*, which encodes the anaerobic glycerol-3-phosphate dehydrogenase (G3PDH) subunit A (Figs 2 and 3A; Table S2). *glpB* and *glpC* coding for subunits B and C of the anaerobic G3PDH, as well as *glpD* coding for the aerobic G3PDH, were also upregulated. Both type of G3PDHs catalyze the reversible conversion of glycerol-3-phosphate to glycerone-3-phosphate^42^. In the absence of glycerol as substrate in the medium, these enzymes participate mainly in the biosynthesis or in the recycling of the main component of cell membrane, glycerophospholipid, *via* the precursor glycerol-3-phosphate^43^. GlpD and GlpABC have been shown in the past to be involved in other stress responses including tolerance to solvents such as butanol and hexane as well as in the formation of persister cells resistant to antibiotics^44–47^. Interestingly, *glpA* transcript abundance was significantly lower in wt grown under UPA230 than SLA (Tables S1 and S3). This suggested that one of the beneficial impacts of the RpoB mutation in T56-1 is to increase *glpA* expression under atmospheric pollution.

Other genes involved in glycerophospholipid metabolism had higher transcript abundance in the adapted strain T56-1 (Figs 2 and 3B). This included genes coding for the glycerol-3-phosphate transporter GlpT, the aquaglyceroporin GlpF and the glycerol kinase GlpK, which catalyzes the reversible conversion of glycerol into glycerol-3-phosphate^48^. Furthermore, six genes coding for proteins involved in the degradation pathway of major bacterial glycerophospholipids also had higher transcript abundance in the adapted strain. This included *glpQ*, which encodes a periplasmic glycerophosphoryl diester phosphodiesterase, *eutDPQT*, which encodes proteins involved in the ethanolamine utilization pathway as well as *eutS*, which encodes a shell protein part of the ethanolamine-degrading metabolosome structure (Figs 2 and 3B)^49–51^. Previously, *glpT*, *glpF*, and *glpQ* were all shown to have higher expression in an *E*. *coli* strain more tolerant to hexane, suggesting that molecular mechanisms involved in the tolerance to solvents may also play a role in the resistance to polluted atmosphere^45^.

Transcriptomic data suggested that the fatty acid metabolism of T56-1 was also altered, which may have an impact on membrane lipids homeostasis and recycling (Figs 2 and 3C). Six genes coding for enzymes involved in fatty acid degradation had higher transcript abundance in the adapted strain. This includes FadD, which participates in the activation of fatty acids released from the membrane lipids, and FadABEHJ, which are involved in the β-oxidation of fatty acids^52,53^. The fatty acid composition of T56-1 and wt was investigated under SLA, where both strains had a similar growth rate to avoid bias due to the correlation between growth rate and fatty acid biosynthesis that could arise under DEA613 (Table S4)^54^. T56-1 had a slightly lower unsaturated-to-saturated fatty acid ratio of 0.77 compared to 0.93 for wt. In response to different stresses, *E*. *coli* was shown to adjust its fatty acid composition^55^. For instance, *E*. *coli* decreased its unsaturated-to-saturated fatty acids ratio when exposed to long-chain solvents^56^.

### Transporters and other cell membrane proteins

Beside glycerophospholipid-related genes, transcript abundance of genes coding for transporters and other cell membrane proteins were significantly different in the adapted strain T56-1 compared to wt under DEA613 (Fig. 2 and Table S5). 24 genes coding for proteins associated with sugar transport and 22 genes linked to the transport of other molecules were upregulated. Among these, higher transcript abundance for the arsenical efflux pump membrane protein ArsB was probably related to the fact that diesel exhaust is a source of arsenic released in the atmosphere^57^. Notably, both *uhpT* coding for an hexose phosphate transporter, and *oxlT* coding for an oxalate/formate antiport family MFS transporter capable of generating a proton motive force (PMF), were upregulated in T56-1 compared to wt grown under DEA613 but were downregulated in UPA230-grown wt versus SLA-grown wt^58,59^. *mglA*, which encodes a galactose/methyl galactoside import ATP-binding protein, had higher transcript in DEA-grown T56-1 and also in DEA-grown wt versus SLA-grown wt (Tables S1 and S6). Only four genes coding for transporter were downregulated in T56-1. 15 other genes coding for membrane proteins were differentially-expressed in the adapted strain. The most notable ones were the downregulated outer membrane protease-coding *ompT* and the porin-coding *ompC*. Interestingly, *ompT* had higher transcript abundance in the DEA-grown wt than in the SLA-grown wt, and *ompC* had lower transcript abundance when the wt was grown under UPA230 compared to SLA. These results suggested that OmpT and OmpC may be detrimental for cells exposed to polluted atmosphere and that T56-1 may benefit from reducing their expression.

Among upregulated genes in the adapted strain T56-1 coding for transporter subunits or other membrane proteins, *uhpT* as well as *mglB* coding for a galactose ABC transporter substrate-binding protein, *malKEFGM* coding for subunits of maltose transporters and *srlA* coding for a glucitol/sorbitol permease IIC component were also shown to be overexpressed in *E*. *coli* in response to solvent exposure^45,60^. In contrast, two genes downregulated in T56-1, *ompT* and *cirA*, which codes for the colicin I receptor, had lower expression after solvent exposure. Additionally, upregulated genes in T56-1 *tauABC* coding for a taurine ABC transporter and *ssuC* coding for an aliphatic sulfonate ABC transporter permease were also shown to have higher expression in an evolved *E*. *coli* strain that became osmotolerant^61^. These observations further suggest similitudes between cell mechanisms responsible for tolerance to atmospheric pollution, solvent and osmotic stresses.

Another tendency exposed by the transcriptomic data was the downregulation of 12 genes coding for hydrogenases 1 and 2, the formate hydrogenlyase and the formate dehydrogenase (Fig. 2 and Table S7). The reason for this differential expression is not clear since there is no link known between atmospheric pollution and the regulation of genes coding for these membrane-associated enzymes. Still, changes in their expression as well as in the abundance of transporters and other membrane proteins in T56-1 could impact on cell membrane structure. To provide additional evidences of change in the cell envelope structure of the adapted strain, profile of membrane protein fractions enriched from wt and T56-1 grown under SLA and DEA613 was analyzed by sodium dodecyl sulfate polyacrylamide gel electrophoresis (SDS-PAGE) (Fig. S3). Interestingly, compared to wt under SLA, *E*. *coli* wt exposed to DEA613 resulted in an overall decrease of cell envelope proteins accumulation, while the exposure of T56-1 to DEA613 had no discernable impact. Other significant membrane protein profile differences between wt and T56-1 include the presence of an intense protein band of *ca*. 100 kDa in T56-1 samples grown under either SLA or DEA613. When combined with the significant alterations observed for genes of the glycerophospholipid metabolism, it is likely that those changes modified membrane properties and conferred an advantage to the adapted strain when interacting with PM or other pollutants found in DEA613.

### Outer membrane permeability

To evaluate if alterations in glycerophospholipid metabolism, in membrane protein expression and accumulation had an impact on the properties T56-1 cell envelope, the outer membrane (OM) permeability of wt and T56-1 was evaluated with the hydrophobic fluorescent probe N-phenyl-1-naphthylamine (NPN) (Fig. 3D)^62^. This probe, which is more fluorescent in a hydrophobic environment such as the inside of a biological membrane, cannot enter OM unless it is damaged or made more permeable^63^. Cultures grown under DEA613 compared to SLA had higher fluorescence for both wt and T56-1, showing that pollutants from DEA613 increased the permeability of the OM. When wt was compared to T56-1, significantly higher fluorescence intensity was observed under DEA613 indicating that the cell membrane of the adapted strain became more resistant to pollutant-triggered damages.

### Modification of the extracellular matrix

**Figure 4:**
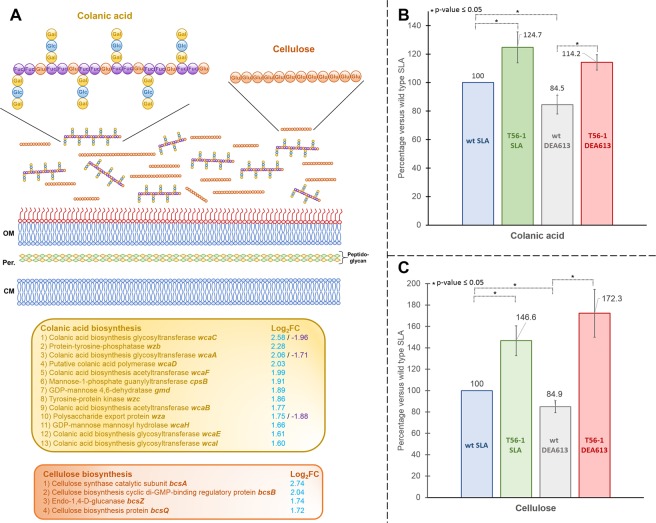
Exopolysaccharides accumulation in the DEA613-adapted strain T56-1. (**A**) Differential expression of genes involved in colanic acid and cellulose biosynthesis. Log_2_FC values represent adapted T56-1 versus wt both grown under DEA613 (blue), and UPA230-grown wt versus SLA-grown wt (purple), respectively. (**B**) Colanic acid accumulation in both adapted strain T56-1 and wt grown under SLA or DEA613. To measure colanic acid, concentration of fucose, which is specific to the colanic acid monomer, was evaluated in the exopolysaccharide fraction^94^. (**C**) Cellulose accumulation in both adapted strain T56-1 and wt grown under SLA or DEA613. Cellulose in the exopolysaccharide fraction was digested with cellulase and the resulting glucose was measured^96^. Colanic acid and cellulose results were normalized with OD 600 nm and are the mean and standard deviation of three replicates. Fuc: fucose, Glu: glucose, Gal: galactose, Glc: glucuronic acid.

RNA sequencing results indicated that the three genes *wza*, *wzb* and *wcaA*, which participate to the metabolism of the capsular exopolysaccharides colanic acid, had lower transcript abundance in the UPA230-grown wt versus SLA-grown wt (Fig. 4A and Table S1). On the contrary, the transcriptome of the adapted strain T56-1 indicated that 13 genes found in the *wca* cluster involved in the colanic acid metabolism had higher transcript abundance (Fig. 4A and Table S8). The *wca* cluster comprises 20 genes coding for proteins responsible for the polymerization of glucose, galactose, fucose and glucuronic acid repeats, which form colanic acid^64^. Upregulated genes included *gmd*, *fcl*, *wcaH*, *cpsB* and *wcaI*, which are involved in GDP-fucose synthesis and transfer, *wcaA*, *wcaC* and *wcaE*, which are responsible for the sequential assembly of other sugar monomers, and *wza*, *wzb*, and wzc, which engage in colanic acid polymerization and translocation from the inner membrane to the outer membrane surface^65,66^.

Colanic acid is thought to participate in the formation of mature biofilm in *E*. *coli* and its synthesis has been shown to be linked to cell envelope damage as well as stresses such as osmotic shock, rapid acid shift and oxidative stress^67–69^. It is not clear how atmospheric pollutants from UPA230 interact with the regulatory network of *E*. *coli* wt to inhibit the expression of colanic acid-related genes. However, a clear observation is that adaptation to DEA613 resulted in higher expression of most genes of the wca cluster highlighting the likely importance of colanic acid for exposure to heavy atmospheric pollution. Results presented here on the *wca* cluster also constitute a good example of how living cells can become fitter under multifactorial stresses such as atmospheric pollution by the acquisition of a single mutation altering the expression of multiple genes including critical ones.

Four genes, *bcsA*, *bcsB*, *bcsQ* and *bcsZ* involved in the metabolism of the exopolysaccharide cellulose, had higher transcript abundance in the adapted strain T56-1 (Fig. 4A and Table S8). Exopolysaccharide cellulose is one of the major constituents of the *E*. *coli* extracellular matrix. Cellulose participates in cell resistance to several stresses such as desiccation, bleach, low temperature, low nutrient and H_2_O_2_^70^. The upregulated genes include *bcsA*, which encodes the membrane-embedded cellulose synthase responsible for polymerizing UDP-D-glucose monomers into cellulose chain, and *bcsB*, which encodes the co-catalytic membrane protein accompanying BcsA^71^. BcsZ is an extracellular endo-1,4-D-glucanase possibly involved in cellulose production proofreading, and BcsQ is a cellulose biosynthesis protein^72^. Interestingly, the most downregulated gene in T56-1 was *pyrB*, which encodes the aspartate carbamoyltransferase catalytic subunit involved in pyrimidine biosynthesis and amino acid metabolism (Table S9). This enzyme catalyzes the conversion of carbamoyl-phosphate into n-carbomoyl-aspartate, a compound that inhibits the diguanylate cyclase DgcQ, which activates cellulose production *via* the second messenger c-di-GMP^73^.

To evaluate the impact of higher expression of colanic acid-related genes and cellulose-related genes on the extracellular matrix of T56-1, accumulation of both exopolysaccharides was measured (Fig. 4B,C). T56-1 accumulated more colanic acid and cellulose than wt under both SLA and DEA613. Furthermore, DEA613-grown wt had significantly less colanic acid and cellulose than SLA-grown wt, which matched the transcriptomic results in the case of colanic acid and provided more evidence supporting a relation between exopolysaccharides and *E*. *coli* reaction to heavily polluted atmosphere.

### Tryptophan and other amino acids

**Figure 5:**
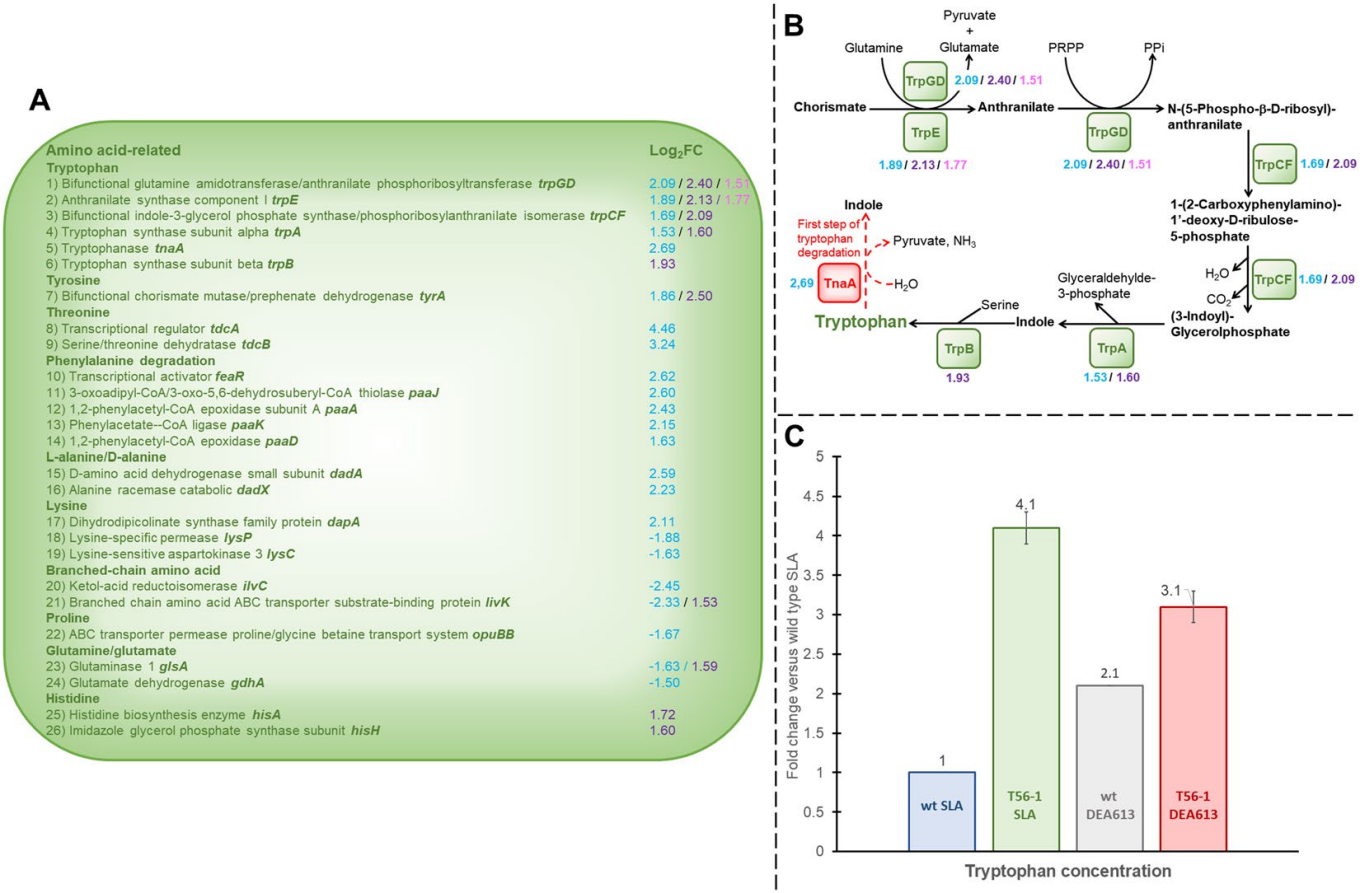
Amino acids metabolism in the DEA613-adapted strain T56-1. (**A**) Differential expression of genes involved in amino acids metabolism. (**B**) The tryptophan biosynthesis pathway. Log_2_FC values in blue, purple and pink represent adapted T56-1 versus wt both grown under DEA613, UPA230-grown wt versus SLA-grown wt, and DEA613-grown wt versus SLA-grown wt, respectively. (**C**) Tryptophan concentration fold change in both adapted strain T56-1 and wt grown under SLA or DEA613. Tryptophan concentration results were normalized with OD 600 nm and are the mean and standard deviation of three replicates.

When comparing UPA230-grown wt or DEA613-grown wt to SLA-grown wt, and comparing DEA613-grown T56-1 to DEA613-grown wt, only two genes always showed higher transcript abundance, which were *trpE* and *trpGD*. *trpE* is a gene coding for the anthranilate synthase component I, and *trpGD* encodes for the bifunctional glutamine amidotransferase/anthranilate phosphoribosyltransferase (Fig. 5A,B, Tables S1 and S10)^74^. Both genes are involved in the biosynthesis of the amino acid tryptophan. Other genes participating in the tryptophan metabolism were upregulated in the adapted strain including *tnaA*, which encodes a tryptophanase converting tryptophan to indole, as well as *trpCF* and *trpA*, which encodes the bifunctional indole-3-glycerol phosphate synthase/phosphoribosylanthranilate isomerase and the tryptophan synthase subunit alpha, respectively. Both *trpCF* and *trpA* also had higher transcript abundance in the wt grown under UPA230 versus SLA (Fig. 5A,B and Table S1). *trpB*, which encodes the tryptophan synthase subunit beta, was another tryptophan-related gene upregulated when wt was exposed to UPA230. Tryptophan has been shown to be involved in ethanol stress response in *E*. *coli* and yeast as well as in mercury resistance in rice where it reduced mercury-induced production of ROS^75,76^.

Tryptophan concentration was measured during the exponential growth phase in wt and in T56-1 to assess if the differential expression of tryptophan metabolism-associated genes had a physiological impact (Fig. 5C). Tryptophan concentration was doubled when the wt was grown under DEA613 compared to SLA. In the adapted strain T56-1, tryptophan concentrations were significantly higher than in wt under either DEA613 or SLA atmosphere. These results agreed with the transcriptomic data since DEA613 caused an upregulation of *trpGD* as well as *trpE* in the wt and the adaption process augmented further the expression of these genes.

Multiple genes related to amino acids other than tryptophan were differentially-expressed in the adapted strain T56-1 (Fig. 5A and Table S10). This includes the upregulation of genes associated with the metabolism of threonine, alanine, lysine, tyrosine as well as the downregulation of genes related to branched amino acids, lysine, proline, glutamate and glutamine. Additionally, five genes participating in phenylalanine degradation had higher transcript abundance. Two genes that were downregulated in T56-1 had higher transcript abundance in wt exposed to UPA230: *livK*, which encodes a branched-chain amino acid ABC transporter substrate-binding protein, and *glsA*, which codes for the glutaminase 1. Two histidine synthesis-related genes were also upregulated in wt grown under UPA230 (Tables S1 and S3). Alteration of the expression of genes coding for enzymes involved in amino acids metabolism including tryptophan may be a response to an increase in proteins damaged by atmospheric pollutants. These proteins must be recycled and replaced by new ones^77^. In the same vein, the high number of upregulated genes coding for transporters and other membrane proteins may also be a response to replace damaged proteins.

### Response to reductive electrophilic species

**Figure 6:**
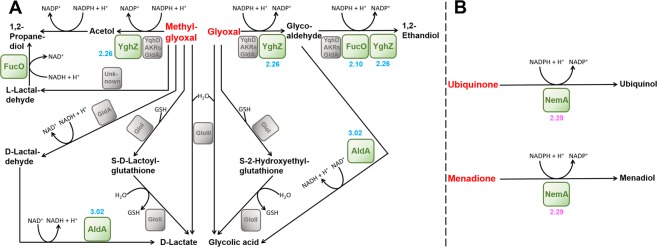
Differential expression of genes involved in the detoxification of RES in *E*. *coli*. (**A**) (Methyl) glyoxals^12^ and (**B**) quinones detoxification. Log_2_FC values in blue and pink represent adapted T56-1 versus wt both grown under DEA613, and DEA613-grown wt versus SLA-grown wt, respectively.

Three genes, *fucO*, *aldA* and *yghZ* associated with (methyl)glyoxal detoxification had higher transcript abundance in T56-1 versus wt under DEA613 (Fig. 6A and Table S11). *fucO*, which is also involved in fucose degradation, codes for a lactaldehyde reductase catalyzing the conversion of glycolaldehyde to 1,2-ethanediol within the glyoxal pathway and the conversion of l-lactaldehyde to 1,2-propanediol within the methylglyoxal pathway. *yghZ* codes for a glyceraldehyde 3-phosphate reductase that can convert methylglyoxal into acetol, glyoxal into glycoaldehyde and glycoaldehyde into 1,2-ethandiol^78^. *aldA* codes for an aldehyde dehydrogenase catalyzing the generation of glycolic acid from glycolaldehyde in the glyoxal pathway and the generation of D-lactate from D-lactaldehyde in the methylglyoxal pathway. Interestingly, *aldB*, which codes for another aldehyde dehydrogenase was also upregulated in the adapted strain (Table S11). Beside (methyl)glyoxal, severe atmospheric pollution such as DEA613 contains other toxic aldehydes such as acrolein, formaldehyde and crotonaldehyde, which may trigger the expression of several aldehyde dehydrogenases capable of oxidizing a large range of substrates^79^.

When comparing wt grown under DEA613 to wt grown under SLA, the most upregulated gene was *nemR*, which encodes a transcriptional regulator involved in the cell response to RES (Fig. 6B and Table S6). *nemR* is located on the same operon as *nemA* and *gloA*, which encodes a N-ethylmaleimide reductase and the glyoxalase I, respectively. Of these two genes, only *nemA* was significantly upregulated after exposure of wt to DEA613. NemR, which is a repressor, dissociates itself from its own promoter upon sensing quinones and glyoxals leading to higher expression of the *nemR-nemA-gloA* operon. Beside N-ethylmaleimide, NemA also reduces quinones and participates in their detoxification^80^. Interestingly, *cydAB*, which encodes two subunits of the cytochrome bd-II ubiquinol oxidase had lower transcript abundance in T56-1 compared to wt under DEA613 (Fig. 2). This enzyme is a component of *E*. *coli* aerobic respiratory chain involved in PMF generation and it couples ubiquinol oxidation into ubiquinone with O_2_ reduction. Its downregulation may be related to the presence of toxic reactive quinones in cells exposed to DEA613.

## Discussion

In ecosystems with heavy atmospheric pollution such as major urban centers and industrial area, organisms are exposed to a multifactorial stress that may result in the fixation of adaptive mutations in their genome. Here, we showed that the bacterium *E*. *coli* acquired a single mutation in *rpoB* after exposure to atmospheric pollutants. In ALE experiments, mutations in enzymes involved in global transcription such as RpoB are often the first beneficial ones to be fixed, because they usually grant major fitness advantage *via* their impact on the expression of multiple genes^81^. It has been suggested that this type of mutation has a restorative effect by returning the cells to a pre-stressed physiology^37^. Results presented here displayed several examples of this phenomenon. For instance, gene expression in the *wca* cluster as well as colanic acid accumulation, which were reduced in WT exposed to polluted air, were brought back to higher level in the adapted strain. Expression of *ompT*, which was upregulated in wt exposed to atmospheric pollution, was brought down in T56-1. In other case, the adaptation process caused an amplification of the regulatory response instead. For instance, genes associated with tryptophan metabolism were upregulated when exposing wt to air pollutants and had an even higher expression after adaptation.

To further confirm the impact of RpoB R12L on *E*. *coli*’s physiology, it would have been informative to reconstruct an *E*. *coli* BW25113 strain carrying the SNP responsible in its genome by genetic engineering. Unfortunately, a limitation of the study presented here is that attempts at engineering a BW25113 strain with RpoB R12L *via* different methods including CRISPR-Cas9 failed, which suggested that this specific mutation may be problematic for genome editing in *E*. *coli*.

Usually during adaptation to a single stress, cells will acquire other beneficial mutations with lesser impact after the first ones^24,34^. Here, the beneficial mutation rate was slower since only one mutation was detected after 56 transfers when the growth rate became stabilized. It is also possible that the presence of RESs, ROSs, PMs and other sources of cellular stress in DEA613 is responsible for this phenomenon. Under these circumstances, mutations that would be beneficial in response to a given stress may be detrimental for another stress.

In itself, atmospheric pollution has mutagenic effects on living cells^82^. Besides RES, other compounds such as metal, PAHs, ROS and RNS also can damage DNA or impede DNA repair and synthesis system^8^. The genotoxic effect of these compounds may augment the incidence of different types of cancer. For instance, long-term exposure to heavy atmospheric pollution increases lung cancer risk by 10–30%^83,84^. Results presented here indicated that adaptive mutations that could be spontaneous instead of chemically-driven must also be considered when investigating the genome-modifying effect of atmospheric pollution. It is not clear if the *rpoB* mutation was caused by a random event or by genotoxic compounds found in DEA613. Based on the low rate of fixed mutation in T56-1 genome compared to other ALE experiments, it cannot be concluded that DEA613 has significant mutagenic property. Further study would be required to provide more insights on the relation between mutagenicity and atmospheric pollution.

The *E*. *coli* response to heavy atmospheric pollution described here involved multiple cell functions such as change in the membrane properties, in exopolysaccharides accumulation and in amino acid concentration. After adaptation, it appears that the bacterium benefits from significantly modifying its cell wall structure to either minimize damages caused by atmospheric pollutants or to be able to make quick reparation. Exposure to atmospheric pollution triggered permanent genetic alteration with considerable impact on the cell physiology. This further demonstrates the repercussion of human activity *via* the acquisition of adaptive mutations on the evolution of environmental microorganisms and other living cells. It is also possibly representative of the changes materializing in higher organisms constantly exposed to atmospheric pollution.

## Methods

### Strains, growth medium and atmosphere

*E*. *coli* BW25113^85^ was grown and maintained at 37 °C in M9 minimal medium containing glucose (4 g l^−1^), NaCl (0.5 g l^−1^), NH_4_Cl (1 g l^−1^), Na_2_HPO_4_ (47.8 mM), KH_2_PO_4_ (22 mM), MgSO_4_ (2 mM), and CaCl_2_ (0.1 mM). For growth under SLA, UPA230 or DEA613, sealed anaerobic tubes or serum bottles containing 10 ml or 100 ml M9-glucose medium were flushed with a specific atmosphere prior to inoculation. UPA230 was collected from the campus of Wuhan University of Technology on December 4^th^ 2017. DEA613 was sampled from the exhaust of a XP8500XE-3D diesel generator (Xingpu, China). UPA230 composition was obtained from the Wuhan Environmental Protection Bureau (China). DEA613 composition was evaluated with a BR-Smart-121 PM_2.5_ and PM_10_ detector (Bolangtong, China) and an ADKS-4 SO_2_, NO_2_, O_3_ and CO gas detector (EDKORS, China).

### Adaptive laboratory evolution under DEA613

ALE experiment was started with an overnight M9-glucose culture under SLA initiated with cells from a *E*. *coli* BW25113 freezer stock. The first transfer with a 10% inoculum was done in duplicate into anaerobic tubes containing 10 ml medium under DEA613. And then, two independent series of cultures were transferred serially into new tubes more than 56 times with 10% inoculum once the cultures reached the mid-log growth phase. Only one set of the two series showed significantly improved growth rate in M9-glucose medium under DEA613 after 56 transfers, and thus it was chosen for subsequent experiments. Samples were frozen periodically with 25% glycerol and stored at −80 °C for future use. T56 culture was streaked on three separated M9-glucose plates and one clone was isolated from each plate. Identities of the clones were verified by PCR.

### Scanning electron microscopy

*E*. *coli* cultures were filtered with 0.22 μm polyethersulfone filters, and the bacteria-covered filters were incubated overnight in a buffer solution (0.1 M phosphate buffer, PH 7) containing 2.5% glutaraldehyde at 4 °C. The samples were dehydrated with solutions of increased ethanol concentration (30 to 90% ethanol), and then they were further dehydrated with two 100% ethanol washes. Air-dried samples were observed with a JEOL-7100F scanning electron microscope at an accelerating voltage of 20 kV.

### Whole genome DNA sequencing

Genomic DNA was extracted with M5 Bacteria Genomic DNA Kit (Mei5 Biotechnology, China) from 3 ml of T56-1 and T56-3 M9-glucose cultures. The genomic libraries were generated with NEB Next Ultra DNA Library Prep Kit for Illumina (NEB, MA). After ligation of the adapters, DNA fragments ranging from 300 to 400 bp were recovered by beads purification. The adapter-modified DNA fragments were enriched by twelve cycles-PCR. Sequencing was done with an Illumina HiSeq 4000 (Illumina, CA) with a paired-end protocol and read lengths of 150 nucleotides. The sequencing reads were then trimmed with PRINSEQ before being aligned and used for variant calling with the Picard DNA-seq analysis pipeline and SAMtools^86–88^. The reference genome for the analysis was *Escherichia coli* BW25113 (NCBI reference sequence NZ_CP009273.1). All the samples had an average coverage of at least 30X.

### RNA sequencing

*E*. *coli* wt grown under SLA, UPA230 and DEA613 as well as adapted strain T56-1 grown under DEA613 were cultivated in triplicate in 100 ml M9-glucose medium. After reaching mid-log phase, cultures were snap-freezed in liquid nitrogen and total RNA was extracted with the TRIzol reagent (ThermoFisher Scientific, MA). A Ribo-Zero rRNA Removal Kit for bacteria (Illumina) was applied to deplete RNAs present in the total RNA samples. Sequencing libraries were prepared with the TruSeq RNA sample preparation kit (Illumina). Sequencing was conducted with an Illumina HiSeq 4000 instrument with a paired-end protocol and read lengths of 150 nucleotides. Reads were mapped on the reference *E*. *coli* BW25113 genome with Bowtie 2^89^. Fragments per kilobase of transcript per million mapped reads (FPKM) were calculated with RSEM^90^. FDR-adjusted p-values (q-values) were calculated with EdgeR^91^.

### Fatty acid analysis

*E*. *coli* wt and T56-1 cultures were grown to mid-log phase under SLA. Cells were then harvested by centrifugation at 4 °C, washed twice in phosphate buffered saline, and freeze dried. Samples were sent to Sci-tech innovation company (Qingdao, China), where membrane fatty acids were extracted, transesterified, and analyzed by gas-liquid chromatography.

### Cell envelope protein profiling

Cell envelope proteins were enriched and resolved by SDS-PAGE as described previously^92^. Briefly, *E*. *coli* wt and T56-1 cultures were grown to mid-log phase under SLA or DEA613. 2 × 10^9^ cells were harvested by centrifugation, washed with phosphate-buffered saline, and resuspended in 10 mM sodium phosphate buffer (pH 7.2). Cell suspensions were then sonicated and centrifuged to remove unbroken cells and cell debris. Whole-cell lysates were ultracentrifuged 1 hour at 100000 × g at 4 °C. Cell envelope pellets were then washed, mixed with SDS-PAGE loading buffer, incubated five minutes at 95 °C and loaded on 12.5% SDS-PAGE. After electrophoresis, gels were stained with GelCode Blue stain reagent (ThermoFisher Scientific). The ImageJ software was used to confirm differential protein band intensities among samples.

### Outer membrane permeability assay

OM permeability assay was conducted as described previously^62^. Mid-log phase *E*. *coli* cultures (1 ml) were centrifuged, washed, and resuspended in NaCl 0.85% containing 10 μM NPN. The cell suspension was incubated for five minutes at room temperature and fluorescence was measured with a DS-11 FX spectrofluorometer (DeNovix, DE, USA). Excitation and emission wavelength were set at 375 nm and 435–485 nm, respectively.

### Tryptophan assay

For tryptophan concentration measurement, bacterial cells were prepared as described before^93^. Mid-log phase *E*. *coli* cultures were shock-frozen in liquid nitrogen before being diluted to same cell density with NaCl 0.9%. The cell suspension was then sonicated on ice with a Qsonica sonicator (CT, USA). Cell debris were removed by centrifugation and then macromolecules were discarded from the supernatant with Amicon Ultra centrifugal filters with 3 KDa molecular weight cut-off (MWCO) (Millipore, MA, USA). Tryptophan concentration in the treated supernatants was measured with a Trp ELISA kit (mlbio, China). Values were normalized with cell suspension OD 600 nm.

### Colanic acid and cellulose assays

Colanic acid accumulation was assessed *via* the evaluation of fucose concentration in the exopolysaccharide fraction as described previously^94^. Mid-log phase *E*. *coli* cultures (50 ml) were heated at 100 °C for 15 minutes, cooled-down and centrifuged. 40 ml of the supernatant was mixed with 120 ml of 100% ethanol and incubated overnight at 4 °C. After centrifugation, the pellet was suspended in 5 ml ultrapure water before dialysis (MWCO: 3.5 KDa) for 48 hours against ultrapure water. After freeze-dried, the lyophilizate was suspended in 5 ml of 10% trichloroacetic acid and centrifuged to remove residual polypeptides. The supernatant was dialyzed for five more days, freeze-dried, and resuspended in 1 ml ultrapure water. Fucose concentration in these samples was then measured according to the method developed by Disches and Shettles^95^ with an Evolution 220 UV-visible spectrophotometer (Thermo Fisher Scientific).

Cellulose was assayed as described previously with several modifications^96^. Briefly, stationary-phase *E*. *coli* cultures (50 ml) were harvested by centrifugation after reached an OD 600 nm of 1.5 to 1.6. Stationary-phase cultures were used to ensure that all the glucose provided as substrate has been oxidized by bacteria before starting cellulose digestion. After centrifugation, the supernatant was freeze-dried and then resuspended in 50 mM sodium acetate buffer (pH 5.0) at a concentration of 100 mg ml^−1^. Subsequently, cellulase (28 U ml^−1^) from *Trichoderma reesei* (Sigma-Aldrich, MO, USA) was added and the reaction was incubated for 16 hours at 37 °C. For each sample, a control without cellulase was also incubated simultaneously. Standard curve was made with carboxymethylcellulose (CMC) solutions of known concentration digested by cellulase in the same way as the samples. Glucose present in the cellulase-treated samples, CMC standards or untreated samples were measured with the glucose hexokinase assay kit (Sigma-Aldrich). Both colanic acid-related and cellulose-related values were normalized with cultures OD 600 nm.

## Supplementary information

Supplementary information

## Data Availability

DNA sequencing reads for strains T56-1 and T56-3 are available in the NCBI Sequence Read Archive (SRA) under Accession Number SRP213893. RNA sequencing data have been deposited with the NCBI GEO database under Accession Number GSE115330. The data supporting this study are available from the corresponding authors upon request.
