# Supplementary material for: Escherichia coli adaptation and response to exposure to heavy atmospheric pollution
Source: Sci Rep. 2019 Jul 26;9:10879. doi: 10.1038/s41598-019-47427-7 (PMC6659633; doi:10.1038/s41598-019-47427-7)
Supplement: Supplementary file 1 — Supplementary information [file 41598_2019_47427_MOESM1_ESM.docx]

**Supplementary information**

***Escherichia coli* adaptation and response to exposure to heavy atmospheric pollution**

**Authors:** Tian Zhang*^a,b^, Xiao-Chen Shi*^a,b^, Yangyang Xia^c^, Liqiang Mai^c^, Pier-Luc Tremblay^#a,b^

**^*^**Both authors contributed equally to this work.

**Author affiliation:**

^a^ State Key Laboratory of Silicate Materials for Architectures, Wuhan University of Technology, Wuhan P.R. China

^b^ School of Chemistry, Chemical Engineering and Life Science, Wuhan University of Technology, Wuhan, P.R. China

^c^ State Key Laboratory of Advanced Technology for Materials Synthesis and Processing, International School of Materials Science and Engineering, Wuhan University of Technology, Wuhan, P.R. China

**
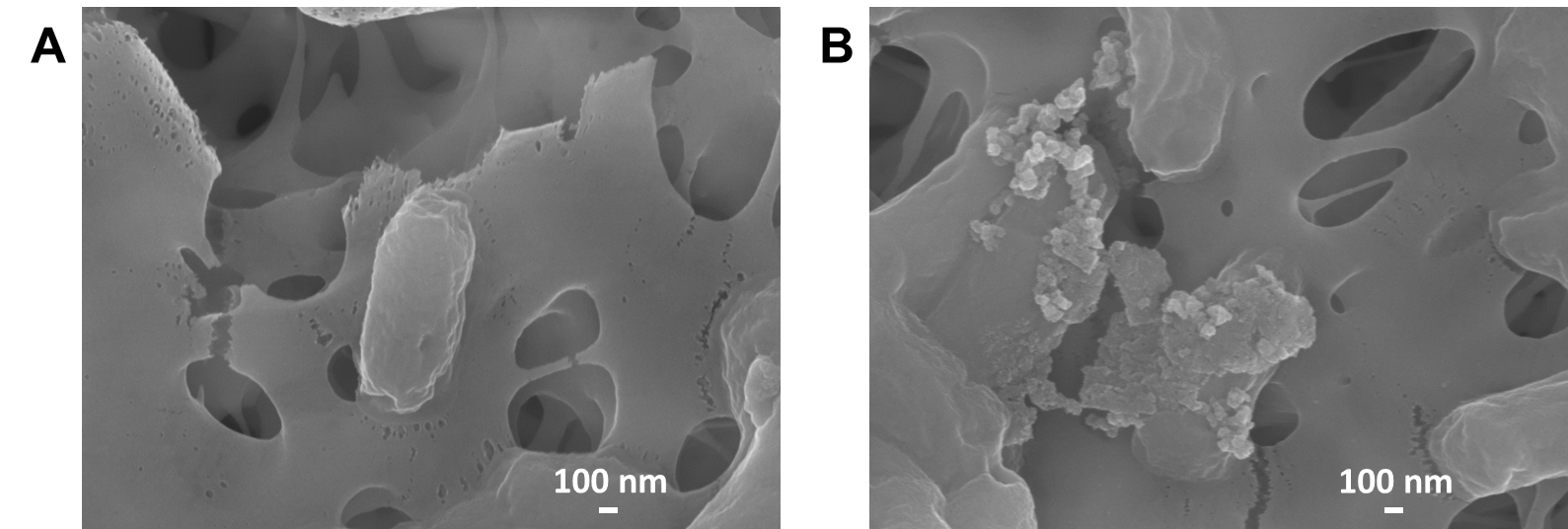
**

**Figure S1.** SEM images of DEA613-adapted strain T56-1 grown under **a** SLA or under **b** DEA613.

**
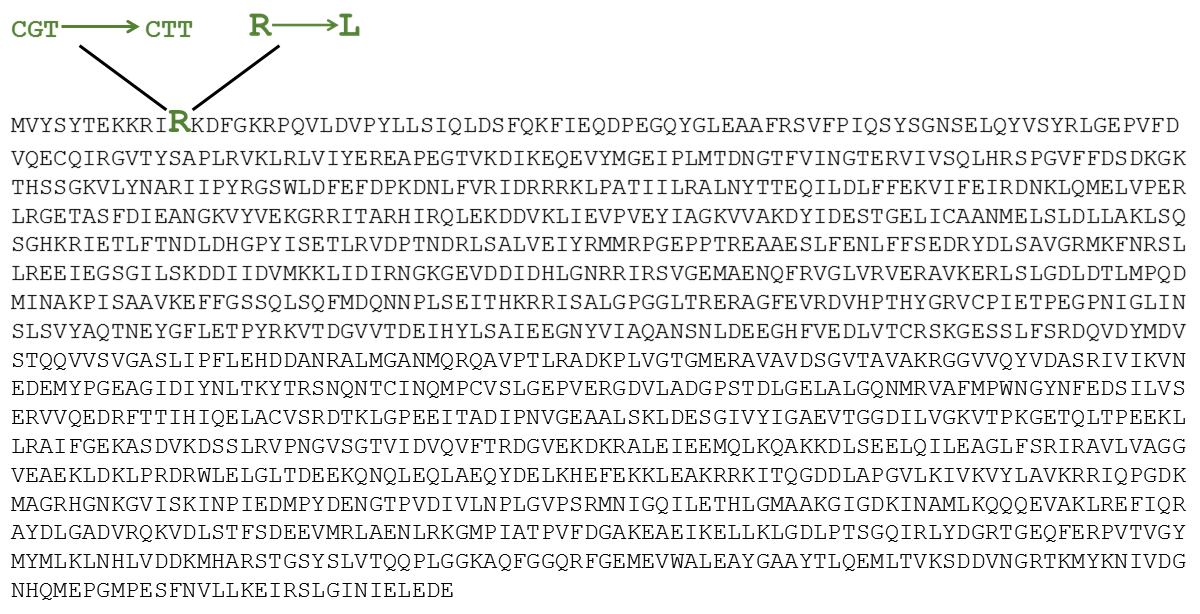
**

**Figure S2.** Protein sequence of *E. coli* BW25113 RNA polymerase β-subunit (WP_000263098.1). Single nucleotide polymorphism of a guanine (G) for a thymine (T) at position 35 of the DNA sequence resulted in the substitution of an arginine (R) for a leucine (L) at position 12 of the protein sequence in the DEA613-adapted T56 strain clone #1 and clone #3.


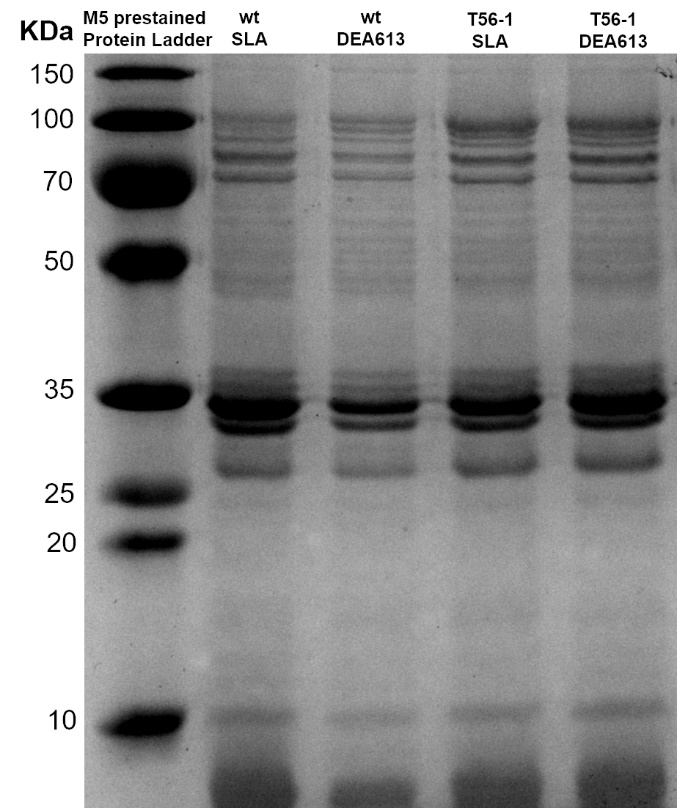


**Figure S3.** SDS-PAGE profile of cell membrane proteins in *E. coli* wt and T56-1 grown under SLA or DEA613. Cell envelopes were isolated from 2 × 10^9^ cells and analyzed by SDS-PAGE. Experiment were done in duplicate to verify results (not shown).

| **Table S1.** Differentially-expressed genes in more than one RNA sequencing experiment.**^a,b,c,d^** | | | | |
| --- | --- | --- | --- | --- |
| **Locus** | **Annotation** | **DEA613-grown WT vs. SLA-grown WT** | **UPA230-grown WT vs. SLA-grown WT** | **DEA613-grown T56-1 vs. DEA613-grown WT** |
| BW25113_RS23775 | Stress response membrane protein YncL | 2.50 | NA**^e^** | -1.85 |
| BW25113_RS06860 | Sucrose phosphorylase | 1.89 | 1.57 | NA |
| BW25113_RS06590 | Anthranilate synthase component I TrpE | 1.77 | 2.13 | 1.89 |
| BW25113_RS16660 | Periplasmic chaperone protein | 1.67 | 1.59 | NA |
| BW25113_RS02925 | Outer membrane protease OmpT | 1.61 | NA | -2.60 |
| BW25113_RS11210 | Galactose/methyl galactoside import ATP-binding protein MglA | 1.60 | NA | 4.34 |
| BW25113_RS05140 | Cold-shock protein CspI | 1.56 | NA | -2.09 |
| BW25113_RS06585 | Bifunctional glutamine amidotransferase/anthranilate phosphoribosyltransferase TrpD | 1.51 | 2.40 | 2.09 |
| BW25113_RS05045 | Hydrogenase-1 small subunit HyaA | -1.60 | NA | -2.43 |
| BW25113_RS18165 | Hypothetical protein | NA | 3.42 | 3.16 |
| BW25113_RS13575 | Bifunctional chorismate mutase/prephenate dehydrogenase TyrA | NA | 2.50 | 1.86 |
| BW25113_RS06580 | Bifunctional indole-3-glycerol phosphate synthase/phosphoribosylanthranilate isomerase TrpF | NA | 2.09 | 1.69 |
| BW25113_RS06570 | Tryptophane synthase subunit alpha TrpA | NA | 1.60 | 1.53 |
| BW25113_RS02505 | Glutaminase 1 GlsA | NA | 1.57 | -1.63 |
| BW25113_RS17915 | Branched chain amino acid ABC transporter substrate-binding protein LivK | NA | 1.53 | -2.33 |
| BW25113_RS05050 | Hydrogenase-1 large chain HyaB | NA | -1.50 | -2.82 |
| BW25113_RS02855 | Porin OmpC | NA | -1.55 | -1.65 |
| BW25113_RS10750 | Colanic acid biosynthesis glycosyltransferase WcaA | NA | -1.71 | 2.06 |
| BW25113_RS14955 | Putative selenium-dependent hydroxylase accessory protein YqeC | NA | -1.77 | 5.53 |
| BW25113_RS10765 | Polysaccharide export protein Wza | NA | -1.88 | 1.75 |
| BW25113_RS09970 | Ferritin FtnA | NA | -1.95 | -2.34 |
| BW25113_RS10760 | Protein-tyrosine-phosphatase Wzb | NA | -1.96 | 2.28 |
| BW25113_RS11175 | Hypothetical protein | NA | -1.98 | -1.60 |
| BW25113_RS18625 | Hypothetical protein | NA | -2.12 | 2.34 |
| BW25113_RS11170 | Hypothetical protein | NA | -2.17 | -1.97 |
| BW25113_RS19045 | Hexose phosphate transporter UhpT | NA | -2.96 | 5.63 |
| BW25113_RS11705 | Anaerobic glycerol-3-phosphate dehydrogenase subunit A GlpA | NA | -3.97 | 6.28 |
| BW25113_RS18405 | Oxalate/formate antiport family MFS transporter OxlT | NA | -4.15 | 3.52 |
| **^a^***E. coli* cultures were grown in triplicate in M9-glucose under SLA, UPA230 or DEA613.  **^b^**cut-off 1: log2 fold change ≤ 1.5 or ≥ 1.5.  **^c^**cut-off 2: q-value ≤ 0.05.  **^d^**Values in green and in red are for upregulated and downregulated genes, respectively.  **^e^**NA: not applicable. | | | | |

| **Table S2.** Differentially-expressed genes related to glycerol-3-phosphate, ethanolamine, fatty acid and glycerophospholipid metabolism in the DEA613-adapted strain T56-1 versus wt both grown under DEA613.**^a,b,c^** | | | | |
| --- | --- | --- | --- | --- |
|  | | **FPKM average** | |  |
| **Locus** | **Annotation** | **wt** | **T56-1** | **Log_2_ fold change** |
| **Glycerol-3-phosphate-related** | | | | |
| BW25113_RS11705 | Anaerobic glycerol-3-phosphate dehydrogenase subunit A GlpA | 8.75 | 687.23 | 6.28 |
| BW25113_RS11700 | Glycerol-3-phosphate transporter GlpT | 9.48 | 523.59 | 5.77 |
| BW25113_RS20375 | Aquaglyceroporin GlpF | 21.19 | 890.37 | 5.39 |
| BW25113_RS11710 | Anaerobic glycerol-3-phosphate dehydrogenase subunit B GlpB | 6.70 | 251.87 | 5.02 |
| BW25113_RS17745 | Aerobic glycerol-3-phosphate dehydrogenase GlpD | 38.93 | 1081.40 | 4.79 |
| BW25113_RS20370 | Glycerol kinase GlpK | 35.11 | 863.49 | 4.62 |
| BW25113_RS11715 | Anaerobic glycerol-3-phosphate dehydrogenase subunit C GlpC | 7.92 | 137.97 | 4.11 |
| BW25113_RS11695 | Glycerophosphoryl diester phosphodiesterase GlpQ | 62.06 | 658.45 | 3.41 |
| **Ethanolamine-related** | | | | |
| BW25113_RS12835 | Ethanolamine utilization protein EutS | 0.17 | 6.47 | 4.63 |
| BW25113_RS12830 | Ethanolamine utilization protein EutP | 1.38 | 10.63 | 2.86 |
| BW25113_RS12820 | Ethanolamine utilization cobalamin adenosyltransferase EutT | 2.19 | 9.47 | 2.06 |
| BW25113_RS12825 | Ethanolamine utilization protein EutQ | 2.97 | 12.63 | 2.05 |
| BW25113_RS12815 | Ethanolamine utilization protein EutD | 2.57 | 9.59 | 1.86 |
| **Fatty acid-related** | | | | |
| BW25113_RS19955 | Fatty acid oxidation complex subunit alpha FadB | 11.37 | 81.68 | 2.83 |
| BW25113_RS16000 | NADPH-dependent 2,4-dienoyl-CoA reductase FadH | 5.09 | 29.14 | 2.49 |
| BW25113_RS01120 | Acyl-CoA dehydrogenase FadE | 25.52 | 87.57 | 1.78 |
| BW25113_RS19950 | Acetyl-CoA C-acyltransferase FadA | 17.7 | 60.8 | 1.77 |
| BW25113_RS09445 | Long-chain-fatty-acid--CoA ligase FadD | 68.95 | 218.78 | 1.66 |
| BW25113_RS12215 | Fatty acid oxidation complex subunit alpha FadJ | 28.12 | 85.96 | 1.61 |
| **^a^***E. coli* cultures were grown in triplicate in M9-glucose medium under DEA613.  **^b^**cut-off 1: log_2_ fold change ≤ 1.5 or ≥ 1.5  **^c^**cut-off 2: q-value ≤ 0.05 | | | | |

| **Table S3.** Differentially-expressed genes in *E. coli* wt grown under UPA230.**^a,b,c^** | | | | |
| --- | --- | --- | --- | --- |
|  | | **FPKM average** | |  |
| **Locus** | **Annotation** | **SLA** | **UPA230** | **Log_2_ fold change** |
| BW25113_RS18455 | Protein HokA | 4.44 | 73.26 | 4.01 |
| BW25113_RS18165 | Hypothetical protein | 0.30 | 4.17 | 3.42 |
| BW25113_RS18035 | Hypothetical protein | 1.38 | 12.07 | 3.04 |
| BW25113_RS24725 | Hypothetical protein | 1.92 | 14.14 | 2.81 |
| BW25113_RS13575 | Bifunctional chorismate mutase/prephenate dehydrogenase TyrA | 306.41 | 1734.56 | 2.50 |
| BW25113_RS24700 | Hypothetical protein | 5.72 | 30.67 | 2.40 |
| BW25113_RS06585 | Bifunctional glutamine amidotransferase/anthranilate phosphoribosyltransferase TrpD | 98.91 | 521.76 | 2.40 |
| BW25113_RS24650 | Hypothetical protein | 5.73 | 29.92 | 2.37 |
| BW25113_RS13580 | Phospho-2-dehydro-3-deoxyheptonate aldolase Tyr-sensitive AroF | 710.22 | 3471.93 | 2.29 |
| BW25113_RS06590 | Anthranilate synthase component I TrpE | 85.19 | 373.69 | 2.13 |
| BW25113_RS08135 | Hypothetical protein | 87.03 | 376.17 | 2.11 |
| BW25113_RS06580 | Bifunctional indole-3-glycerol phosphate synthase/phosphoribosylanthranilate isomerase TrpF | 155.52 | 660.35 | 2.09 |
| BW25113_RS10305 | Metal-binding protein ZinT | 3.02 | 12.35 | 2.00 |
| BW25113_RS09050 | Hypothetical protein | 4.89 | 19.81 | 2.00 |
| BW25113_RS06575 | Tryptophan synthase subunit beta TrpB | 367.86 | 1401.20 | 1.93 |
| BW25113_RS25185 | Hypothetical protein | 17.29 | 64.52 | 1.89 |
| BW25113_RS07650 | Hypothetical protein | 2.92 | 10.94 | 1.87 |
| BW25113_RS21755 | DNA alkylation response protein AidB | 34.65 | 125.81 | 1.86 |
| BW25113_RS23830 | Cold-shock protein | 291.71 | 1048.52 | 1.85 |
| BW25113_RS00705 | Fimbrial-like adhesin protein | 1.01 | 3.87 | 1.83 |
| BW25113_RS06030 | DUF1398 domain-containing protein | 6.04 | 21.80 | 1.83 |
| BW25113_RS05150 | Cold-shock protein | 35.65 | 126.78 | 1.83 |
| BW25113_RS08930 | YdiU family protein | 68.73 | 234.72 | 1.77 |
| BW25113_RS15865 | Hypothetical protein | 57.77 | 194.35 | 1.75 |
| BW25113_RS16790 | Barnase inhibitor YhcO | 23.52 | 78.24 | 1.73 |
| BW25113_RS10225 | DUF2158 domain-containing protein | 139.23 | 458.67 | 1.72 |
| BW25113_RS10575 | 1-(5-phosphoribosyl)-5-((5-phosphoribosylamino)methylideneamino)imidazole-4-carboxamide isomerase/histidine biosynthesis HisA | 329.48 | 1086.71 | 1.72 |
| BW25113_RS00540 | Hypothetical protein | 21.85 | 69.83 | 1.67 |
| BW25113_RS06560 | YciE/YciF family protein | 6.19 | 19.66 | 1.65 |
| BW25113_RS05155 | Protein GnsA | 110.73 | 337.67 | 1.61 |
| BW25113_RS08140 | cold-shock protein CspI | 331.82 | 1012.47 | 1.61 |
| BW25113_RS10570 | Imidazole glycerol phosphate synthase subunit HisH | 346.24 | 1053.09 | 1.60 |
| BW25113_RS06570 | Tryptophan synthase subunit alpha TrpA | 512.08 | 1550.27 | 1.60 |
| BW25113_RS16660 | Periplasmic chaperone protein | 1.99 | 6.21 | 1.59 |
| BW25113_RS02505 | Glutaminase 1 GlsA | 42.94 | 129.18 | 1.59 |
| BW25113_RS06860 | Sucrose phosphorylase | 12.15 | 36.25 | 1.57 |
| BW25113_RS23620 | Hypothetical protein | 192.90 | 571.56 | 1.57 |
| BW25113_RS23580 | Cold-shock protein | 948.34 | 2774.80 | 1.55 |
| BW25113_RS17915 | Branched chain amino acid ABC transporter substrate-binding protein Livk | 160.48 | 463.60 | 1.53 |
| BW25113_RS22740 | DUF1435 domain-containing protein | 691.54 | 2002.85 | 1.53 |
| BW25113_RS18180 | MgtC/SapB family protein MgtC | 171.50 | 489.49 | 1.51 |
| BW25113_RS18200 | transcriptional regulator GadE | 412.57 | 1175.01 | 1.51 |
| BW25113_RS22800 | Uncharacterized metal-dependent hydrolase TatD | 57.38 | 162.06 | 1.50 |
| BW25113_RS24490 | Hypothetical protein | 69.82 | 197.78 | 1.50 |
| BW25113_RS05145 | cold-shock protein CspG | 1616.41 | 4584.18 | 1.50 |
| BW25113_RS18405 | Oxalate/formate antiport family MFS transporter OxlT | 354.09 | 19.88 | -4.15 |
| BW25113_RS11705 | Anaerobic glycerol-3-phosphate dehydrogenase subunit A GlpA | 41.12 | 2.53 | -3.97 |
| BW25113_RS03965 | Molybdate-binding periplasmic protein ModA | 588.61 | 59.47 | -3.30 |
| BW25113_RS21415 | Anaerobic C4-dicarboxylate transporter DcuB | 148.17 | 17.65 | -3.06 |
| BW25113_RS15575 | DUF2623 domain-containing protein | 83.11 | 10.23 | -3.01 |
| BW25113_RS19045 | Hexose phosphate transporter UhpT | 37.28 | 4.70 | -2.96 |
| BW25113_RS08760 | 4Fe-4S ferredoxin | 9.56 | 1.22 | -2.87 |
| BW25113_RS15570 | Hypothetical protein | 70.79 | 10.17 | -2.79 |
| BW25113_RS15560 | Hydrogenase-2 operon protein HybA | 252.39 | 41.36 | -2.61 |
| BW25113_RS15565 | Hydrogenase-2 small chain | 337.39 | 59.70 | -2.50 |
| BW25113_RS21410 | Fumarate hydratase class I, anaerobic FumB | 96.74 | 19.19 | -2.33 |
| BW25113_RS05315 | Hypothetical protein | 63.27 | 14.02 | -2.17 |
| BW25113_RS11170 | Phosphate starvation-inducible protein PhoH | 361.74 | 80.4 | -2.17 |
| BW25113_RS18625 | Hypothetical protein | 21.62 | 4.91 | -2.12 |
| BW25113_RS11175 | CidB/LrgB family autolysis modulator | 236.52 | 59.77 | -1.98 |
| BW25113_RS10760 | Protein-tyrosine-phosphatase Wzb | 10.88 | 2.73 | -1.96 |
| BW25113_RS09970 | Ferritin FtnA | 249.19 | 64.34 | -1.95 |
| BW25113_RS15555 | Ni/Fe-hydrogenase b-type cytochrome subunit | 59.91 | 15.52 | -1.94 |
| BW25113_RS21375 | Transcriptional regulator AdiY | 72.07 | 19.40 | -1.89 |
| BW25113_RS10765 | Polysaccharide export protein Wza | 11.86 | 3.16 | -1.88 |
| BW25113_RS02815 | Hypothetical protein | 5.10 | 1.33 | -1.86 |
| BW25113_RS14955 | Putative selenium-dependent hydroxylase accessory protein YqeC | 13.59 | 3.92 | -1.77 |
| BW25113_RS03230 | Anaerobic C4-dicarboxylate transporter DcuC | 146.49 | 44.02 | -1.73 |
| BW25113_RS07220 | Universal stress protein F UspF | 967.78 | 294.40 | -1.72 |
| BW25113_RS10750 | Molybdenum ABC transporter permease ModB | 12.75 | 3.82 | -1.71 |
| BW25113_RS03970 | Colanic acid biosynthesis glycosyltransferase WcaA | 185.66 | 56.50 | -1.71 |
| BW25113_RS20860 | Peptidase E pepE | 279.02 | 86.33 | -1.69 |
| BW25113_RS20475 | Glycerol dehydrogenase GldA | 297.96 | 97.31 | -1.61 |
| BW25113_RS09190 | Sulfurtransferase SseA | 460.48 | 153.97 | -1.58 |
| BW25113_RS17450 | Porin OmpC | 60.07 | 20.38 | -1.55 |
| BW25113_RS02855 | Nitrite reductase small subunit nirD | 140.53 | 47.94 | -1.55 |
| BW25113_RS15780 | DUF4051 domain-containing protein | 408.88 | 144.97 | -1.50 |
| BW25113_RS05050 | Hydrogenase-1 large chain HyaB | 111.64 | 39.32 | -1.50 |
| **^a^***E. coli* cultures were grown in triplicate in M9-glucose under SLA or UPA230.  **^b^**cut-off 1: log2 fold change ≤ 1.5 or ≥ 1.5  **^c^**cut-off 2: q-value ≤ 0.05 | | | | |

| **Table S4.** Fatty acid composition of *E. coli* wild type and DEA613-adapted strain T56-1 under SLA.**^a^** | | |
| --- | --- | --- |
|  | **Strain** | |
| **Fatty acid** | **wt** | **T56-1** |
| Unsaturated fatty acid (UFA)^a^ | 40.7% | 36.8% |
| Saturated fatty acid (SFA)^b^ | 43.6% | 47.3% |
| Cyclopropane fatty acid (CFA)^c^ | 1.1% | 1.0% |
| ^a^UFA: 16:1, 18:1  ^b^SFA: 16:0, 18:0  ^c^CFA: 17:0 cyclo | | |

| **Table S5.** Differentially-expressed genes coding for transporters and other cell membrane proteins in the DEA613-adapted strain T56-1 versus wt both grown under DEA613.**^a,b,c^** | | | | |
| --- | --- | --- | --- | --- |
|  | | **FPKM average** | |  |
| **Locus** | **Annotation** | **wt** | **T56-1** | **Log_2_ fold change** |
| **Transporter** | | | | |
| BW25113_RS11700 | Glycerol-3-phosphate transporter GlpT | 9.48 | 523.59 | 5.77 |
| BW25113_RS19045 | Hexose phosphate transporter UhpT | 10.13 | 506.17 | 5.63 |
| BW25113_RS11215 | Galactose ABC transporter substrate-binding protein MglB | 23.39 | 1033.23 | 5.46 |
| BW25113_RS20950 | Maltose/maltodextrin import ATP-binding protein MalK | 20.17 | 878.08 | 5.44 |
| BW25113_RS20375 | Aquaglyceroporin GlpF | 21.2 | 890.4 | 5.39 |
| BW25113_RS20940 | Maltose ABC transporter periplasmic protein MalE | 34.53 | 1375.98 | 5.31 |
| BW25113_RS11205 | Galactoside ABC transporter permease MglC | 4.95 | 163.75 | 5.02 |
| BW25113_RS20935 | Maltose ABC transporter permease MalF | 15.13 | 488.63 | 5.00 |
| BW25113_RS20955 | Maltoporin LamB | 29.98 | 857.12 | 4.83 |
| BW25113_RS20930 | Maltose ABC transporter permease MalG | 13.74 | 298.87 | 4.43 |
| BW25113_RS11210 | Galactose/methyl galactoside import ATP-binding protein MglA | 13.20 | 269. 89 | 4.34 |
| BW25113_RS18290 | C4-dicarboxylate ABC transporter | 56.56 | 1126.95 | 4.31 |
| BW25113_RS20960 | Maltose regulon periplasmic protein MalM | 35.79 | 595.44 | 4.05 |
| BW25113_RS18570 | 2,3-diketo-L-gulonate TRAP transporter permease | 0.22 | 4.00 | 3.67 |
| BW25113_RS19485 | Ribose import ATP-binding protein RbsA | 27.85 | 347.66 | 3.64 |
| BW25113_RS18405 | Oxalate/formate antiport family MFS transporter OxlT | 138.02 | 1582.79 | 3.52 |
| BW25113_RS21960 | Predicted sugar transporter subunit | 13.38 | 128.37 | 3.25 |
| BW25113_RS14570 | L-fucose-proton symporter FucP | 2.18 | 20.65 | 3.18 |
| BW25113_RS07930 | Autoinducer 2 import ATP-binding protein LsrA | 4.43 | 36.41 | 3.01 |
| BW25113_RS19490 | ribose ABC transporter permease RbsC | 34.07 | 254.90 | 2.90 |
| BW25113_RS14430 | ABC transporter substrate-binding protein | 4.78 | 35.30 | 2.86 |
| BW25113_RS18510 | D-xylose-binding periplasmic protein XylF | 2.12 | 15.22 | 2.79 |
| BW25113_RS07935 | Autoinducer 2 import system permease LsrC | 2.75 | 19.68 | 2.79 |
| BW25113_RS14065 | Glucitol/sorbitol permease IIC component SrlA | 1.70 | 11.99 | 2.75 |
| BW25113_RS19495 | D-ribose ABC transporter substrate-binding protein RbsB | 48.21 | 320.17 | 2.73 |
| BW25113_RS20105 | MFS transporter | 2.99 | 13.87 | 2.18 |
| BW25113_RS18580 | 2,3-diketo-L-gulonate ABC transporter substrate-binding protein | 0.72 | 3.56 | 2.16 |
| BW25113_RS21965 | Sugar ABC transporter ATPase | 9.32 | 41.50 | 2.14 |
| BW25113_RS18145 | Arsenical efflux pump membrane protein ArsB | 7.36 | 31.98 | 2.10 |
| BW25113_RS01895 | Taurine ABC transporter permease TauC | 21.24 | 90.58 | 2.09 |
| BW25113_RS18715 | L-lactate permease | 21.02 | 82.73 | 1.97 |
| BW25113_RS01885 | Taurine-binding periplasmic protein TauA | 15.68 | 61.32 | 1.96 |
| BW25113_RS21970 | Sugar ABC transporter permease | 4.78 | 18.52 | 1.93 |
| BW25113_RS00645 | PTS Enzyme IIA | 38.51 | 146.04 | 1.92 |
| BW25113_RS18575 | 2,3-diketo-L-gulonate TRAP transporter permease | 2.02 | 7.67 | 1.87 |
| BW25113_RS19155 | Transporter | 37.28 | 134.69 | 1.85 |
| BW25113_RS14070 | Glucitol/sorbitol-specific phosphotransferase enzyme IIB component SrlE | 2.85 | 10.43 | 1.83 |
| BW25113_RS01330 | S-methylmethionine permease MmuP | 47.70 | 163.50 | 1.78 |
| BW25113_RS04845 | Aliphatic sulfonate ABC transporter permease SsuC | 3.56 | 12.40 | 1.77 |
| BW25113_RS22365 | Enzyme IIB component of PTS | 5.49 | 18.82 | 1.76 |
| BW25113_RS01890 | Taurine import ATP-binding protein TauB | 32.02 | 104.60 | 1.70 |
| BW25113_RS18515 | D-xylose ABC transporter ATP-binding protein XylG | 12.48 | 40.08 | 1.68 |
| BW25113_RS21235 | D-allose-binding periplasmic protein AlsB | 8.54 | 26.77 | 1.64 |
| BW25113_RS18105 | MFS transporter | 38.16 | 117.73 | 1.62 |
| BW25113_RS03110 | Carbon starvation protein A | 104.5 | 305.03 | 1.54 |
| BW25113_RS19095 | multidrug transporter EmrD | 19.82 | 56.46 | 1.51 |
| BW25113_RS19600 | Ketol-acid reductoisomerase IlvC | 1048.11 | 191.42 | -2.45 |
| BW25113_RS17915 | Branched chain amino acid ABC transporter substrate-binding protein LivK | 368.22 | 73.05 | -2.33 |
| BW25113_RS10985 | Nickel/cobalt efflux protein RcnA | 171.97 | 36.28 | -2.24 |
| BW25113_RS11255 | Lysine-specific permease LysP | 330.04 | 89.57 | -1.88 |
| BW25113_RS11115 | ABC transporter permease proline/glycine betaine transport system OpuBB | 30.60 | 9.60 | -1.67 |
| **Other membrane proteins** | | | | |
| BW25113_RS21765 | Lipoprotein BsmA | 33.85 | 440.31 | 3.70 |
| BW25113_RS15710 | Conserved inner membrane protein | 0.70 | 9.24 | 3.54 |
| BW25113_RS06275 | Membrane protein | 269.10 | 1598.38 | 2.57 |
| BW25113_RS10195 | Flagellar biosynthesis protein FliR | 17.74 | 101.12 | 2.50 |
| BW25113_RS25330 | Periplasmic protein | 48.82 | 156.05 | 1.67 |
| BW25113_RS18605 | outer membrane protein OmpV | 3.48 | 10.94 | 1.62 |
| BW25113_RS03510 | Chitoporin | 5.55 | 17.18 | 1.61 |
| BW25113_RS02925 | Outer membrane protease OmpT | 1850.52 | 304.41 | -2.60 |
| BW25113_RS05080 | Cytochrome bd-II ubiquinol oxidase subunit 2 CydB | 113.88 | 25.20 | -2.17 |
| BW25113_RS05075 | Cytochrome bd-II ubiquinol oxidase subunit 1 CydA | 137.34 | 30.40 | -2.17 |
| BW25113_RS11250 | Colicin I receptor CirA | 572.08 | 143.02 | -2.00 |
| BW25113_RS02875 | Lipoprotein bor | 67.71 | 18.92 | -1.83 |
| BW25113_RS09155 | TVP38/TMEM64 family membrane protein YdjX | 108.34 | 32.68 | -1.73 |
| BW25113_RS11005 | Fimbrial assembly chaperone protein StcB | 2.44 | 0.68 | -1.70 |
| BW25113_RS02855 | Porin OmpC | 139.15 | 44.26 | -1.65 |
| **^a^***E. coli* cultures were grown in triplicate in M9-glucose medium under DEA613.  **^b^**cut-off 1: log_2_ fold change ≤ 1.5 or ≥ 1.5  **^c^**cut-off 2: q-value ≤ 0.05 | | | | |

| **Table S6.** Differentially-expressed genes in *E. coli* wt grown under DEA613.**^a,b,c^** | | | | |
| --- | --- | --- | --- | --- |
|  | | **FPKM average** | |  |
| **Locus** | **Annotation** | **SLA** | **DEA613** | **Log_2_ fold change** |
| BW25113_RS08630 | Transcriptional regulator NemR | 24.26 | 213.22 | 3.13 |
| BW25113_RS23775 | Stress response membrane protein YncL | 5.98 | 34.23 | 2.50 |
| BW25113_RS08635 | N-ethylmaleimide reductase NemA | 57.54 | 281.43 | 2.29 |
| BW25113_RS12455 | PTS fructose transporter subunit IIB | 2.10 | 8.44 | 1.96 |
| BW25113_RS06860 | Sucrose phosphorylase | 11.25 | 41.95 | 1.89 |
| BW25113_RS06590 | Anthranilate synthase component I TrpE | 78.15 | 265.92 | 1.77 |
| BW25  113_RS16660 | Periplasmic chaperone protein | 1.66 | 5.51 | 1.67 |
| BW25113_RS25175 | Hypothetical protein | 47.80 | 149.17 | 1.64 |
| BW25113_RS02925 | Outer membrane protease OmpT | 608.24 | 1850.52 | 1.61 |
| BW25113_RS11210 | Galactose/methyl galactoside import ATP-binding protein MglA | 4.29 | 13.20 | 1.60 |
| BW25113_RS05140 | Cold-shock protein CspI | 33.60 | 99.57 | 1.56 |
| BW25113_RS10375 | Transcriptional regulator Cbl | 49.96 | 145.33 | 1.54 |
| BW25113_RS06585 | Bifunctional glutamine amidotransferase/anthranilate phosphoribosyltransferase TrpGD | 96.34 | 273.8 | 1.51 |
| BW25113_RS05045 | Hydrogenase-1 small subunit HyaA | 72.44 | 23.84 | -1.60 |
| **^a^***E. coli* cultures were grown in triplicate in M9-glucose under SLA or DEA613.  **^b^**cut-off 1: log_2_ fold change ≤ 1.5 or ≥ 1.5  **^c^**cut-off 2: q-value ≤ 0.05 | | | | |

| **Table S7.** Differentially-expressed genes related to membrane-associated enzymes in the DEA613-adapted strain T56-1 versus wt both grown under DEA613.**^a,b,c^** | | | | |
| --- | --- | --- | --- | --- |
|  | | **FPKM average** | |  |
| **Locus** | **Annotation** | **wt** | **T56-1** | **Log_2_ fold change** |
| BW25113_RS05050 | Hydrogenase-1 large chain HyaB | 37.32 | 5.19 | -2.82 |
| BW25113_RS14175 | Formate hydrogenlyase subunit 2 HycB | 96.83 | 17.93 | -2.43 |
| BW25113_RS05045 | Hydrogenase-1 small subunit HyaA | 23.84 | 4.33 | -2.43 |
| BW25113_RS05055 | Ni/Fe-hydrogenase *b*-type cytochrome subunit HyaC | 27.85 | 5.31 | -2.37 |
| BW25113_RS05060 | Hydrogenase 1 maturation protease HyaD | 51.99 | 11.86 | -2.12 |
| BW25113_RS05065 | Hydrogenase-1 operon protein HyaE | 35.35 | 8.93 | -1.97 |
| BW25113_RS05070 | Hydrogenase-1 operon protein HyaF | 33.96 | 8.62 | -1.97 |
| BW25113_RS14170 | Formate hydrogenlyase subunit 3 HycC | 43.56 | 11.50 | -1.91 |
| BW25113_RS14185 | Formate hydrogenlyase regulatory protein HycA | 107.58 | 28.75 | -1.9 |
| BW25113_RS14120 | Electron transporter HydN | 64.04 | 18.57 | -1.78 |
| BW25113_RS21180 | formate dehydrogenase N subunit alpha, selenocysteine-containing Fdhα | 173.85 | 60.15 | -1.53 |
| BW25113_RS14165 | Formate hydrogenlyase subunit 4 HycD | 30.45 | 10.55 | -1.52 |
| **^a^***E. coli* cultures were grown in triplicate in M9-glucose medium under DEA613.  **^b^**cut-off 1: log_2_ fold change ≤ 1.5 or ≥ 1.5  **^c^**cut-off 2: q-value ≤ 0.05 | | | | |

| **Table S8.** Differentially-expressed genes related to exopolysaccharides in the DEA613-adapted strain T56-1 versus wt both grown under DEA613.**^a,b,c^** | | | | |
| --- | --- | --- | --- | --- |
|  | | **FPKM average** | |  |
| **Locus** | **Annotation** | **wt** | **T56-1** | **Log_2_ fold change** |
| **Cellulose-related** | | | | |
| BW25113_RS18315 | Cellulose synthase catalytic subunit BcsA | 7.36 | 49.77 | 2.74 |
| BW25113_RS18310 | Cellulose biosynthesis cyclic di-GMP-binding regulatory protein BcsB | 9.29 | 38.63 | 2.04 |
| BW25113_RS18305 | Endo-1,4-D-glucanase BcsZ | 9.60 | 32.73 | 1.76 |
| BW25113_RS18320 | Cellulose biosynthesis protein BcsQ | 26.37 | 87.03 | 1.72 |
| **Colanic acid-related** | | | | |
| BW25113_RS10740 | Colanic acid biosynthesis glycosyltransferase WcaC | 4.62 | 28.21 | 2.58 |
| BW25113_RS10760 | Protein-tyrosine-phosphatase Wzb | 5.14 | 25.33 | 2.28 |
| BW25113_RS10750 | Colanic acid biosynthesis glycosyltransferase WcaA | 10.14 | 42.68 | 2.06 |
| BW25113_RS10735 | Putative colanic acid polymerase WcaD | 5.46 | 22.60 | 2.03 |
| BW25113_RS10725 | Colanic acid biosynthesis acetyltransferase WcaF | 6.06 | 24.33 | 1.99 |
| BW25113_RS10700 | Mannose-1-phosphate guanylyltransferase CpsB | 3.32 | 12.72 | 1.91 |
| BW25113_RS10720 | GDP-mannose 4,6-dehydratase Gmd | 11.86 | 44.19 | 1.89 |
| BW25113_RS10755 | Tyrosine-protein kinase Wzc | 9.72 | 35.58 | 1.86 |
| BW25113_RS10745 | Colanic acid biosynthesis acetyltransferase WcaB | 6.53 | 22.50 | 1.77 |
| BW25113_RS10765 | Polysaccharide export protein Wza | 11.45 | 38.80 | 1.75 |
| BW25113_RS10710 | GDP-mannose mannosyl hydrolase WcaH | 7.32 | 23.37 | 1.66 |
| BW25113_RS10730 | Colanic acid biosynthesis glycosyltransferase WcaE | 8.89 | 27.37 | 1.61 |
| BW25113_RS10705 | Colanic acid biosynthesis glycosyltransferase WcaI | 4.81 | 14.84 | 1.60 |
| **^a^***E. coli* cultures were grown in triplicate in M9-glucose medium under DEA613.  **^b^**cut-off 1: log_2_ fold change ≤ 1.5 or ≥ 1.5  **^c^**cut-off 2: q-value ≤ 0.05 | | | | |

| **Table S9.** Differentially-expressed genes related to pyrimidine and purine in the DEA613-adapted strain T56-1 versus wt both grown under DEA613.**^a,b,c^** | | | | |
| --- | --- | --- | --- | --- |
|  | | **FPKM average** | |  |
| **Locus** | **Annotation** | **wt** | **T56-1** | **Log_2_ fold change** |
| **Pyrimidine-related** | | | | |
| BW25113_RS03380 | Pyrimidine-specific ribonucleoside hydrolase RihA | 33.68 | 100.05 | 1.57 |
| BW25113_RS11195 | NAD-dependent dihydropyrimidine dehydrogenase subunit PreT | 6.09 | 17.84 | 1.53 |
| BW25113_RS22045 | Aspartate carbamoyltransferase catalytic subunit PyrB | 780.95 | 34.87 | -4.48 |
| BW25113_RS22040 | aspartate carbamoyltransferase regulatory subunit PyrI | 1112.18 | 62.23 | -4.16 |
| BW25113_RS22050 | PyrBI operon leader peptide | 17.78 | 2.67 | -2.69 |
| BW25113_RS01725 | Cytosine permease CodB | 115.75 | 19.63 | -2.55 |
| BW25113_RS04900 | Dihydroorotate dehydrogenase (quinone) PyrD | 121.46 | 27.72 | -2.13 |
| BW25113_RS00165 | Carbamoyl-phosphate synthase small subunit CarA | 571.51 | 172.57 | -1.73 |
| BW25113_RS05535 | Dihydroorotase PyrC | 375.32 | 114.56 | -1.71 |
| BW25113_RS01730 | Cytosine deaminase CodA | 220.82 | 77.26 | -1.51 |
| BW25113_RS13030 | Uracil phosphoribosyltransferase Upp | 430.57 | 151.22 | -1.51 |
| Purine-related | | | | |
| BW25113_RS20785 | Bifunctional phosphoribosylaminoimidazolecarboxamide  formyltransferase/inosine monophosphate cyclohydrolase PurH | 198.12 | 55.41 | -1.84 |
| BW25113_RS18970 | Xanthine permease XanP | 172.23 | 48.12 | -1.84 |
| BW25113_RS02685 | 5-(carboxyamino)imidazole ribonucleotide mutase PurE | 263.95 | 79.89 | -1.72 |
| BW25113_RS13085 | IMP dehydrogenase GuaB | 510.91 | 160.06 | -1.67 |
| BW25113_RS20780 | Phosphoribosylamine--glycine ligase PurD | 163.69 | 53.13 | -1.62 |
| BW25113_RS02680 | 5-(carboxyamino)imidazole ribonucleotide  synthase PurK | 302.05 | 101.87 | -1.57 |
| **^a^***E. coli* cultures were grown in triplicate in M9-glucose medium under DEA613.  **^b^**cut-off 1: log_2_ fold change ≤ 1.5 or ≥ 1.5  **^c^**cut-off 2: q-value ≤ 0.05 | | | | |

| **Table S10.** Differentially-expressed genes involved in amino acids metabolism in the DEA613-adapted strain T56-1 versus wt both grown under DEA613.**^a,b,c^** | | | | |
| --- | --- | --- | --- | --- |
|  | | **FPKM average** | |  |
| **Locus** | **Annotation** | **wt** | **T56-1** | **Log_2_ fold change** |
| BW25113_RS16180 | Transcriptional regulator TdcA | 1.59 | 36.99 | 4.46 |
| BW25113_RS16175 | Serine/threonine dehydratase TdcB | 1.22 | 12.42 | 3.24 |
| BW25113_RS19280 | Tryptophanase TnaA | 3.43 | 22.72 | 2.69 |
| BW25113_RS07265 | Transcriptional activator FeaR | 8.13 | 50.46 | 2.62 |
| BW25113_RS07330 | 3-oxoadipyl-CoA/3-oxo-5,6-dehydrosuberyl-CoA thiolase PaaJ | 14.42 | 88.25 | 2.60 |
| BW25113_RS06190 | D-amino acid dehydrogenase small subunit DadA | 169.06 | 1018.72 | 2.59 |
| BW25113_RS07285 | 1,2-phenylacetyl-CoA epoxidase subunit A PaaA | 1.74 | 9.79 | 2.43 |
| BW25113_RS06195 | Alanine racemase catabolic DadX | 109.91 | 515.11 | 2.23 |
| BW25113_RS07335 | phenylacetate--CoA ligase PaaK | 14.55 | 64.74 | 2.15 |
| BW25113_RS01375 | Dihydrodipicolinate synthase family protein DapA | 4.90 | 21.53 | 2.11 |
| BW25113_RS06585 | Bifunctional glutamine amidotransferase/anthranilate phosphoribosyltransferase TrpGD | 273.80 | 1165.50 | 2.09 |
| BW25113_RS06590 | Anthranilate synthase component I TrpE | 265.92 | 984.36 | 1.89 |
| BW25113_RS13575 | bifunctional chorismate mutase/prephenate dehydrogenase TyrA | 440.93 | 1600.46 | 1.86 |
| BW25113_RS06580 | Bifunctional indole-3-glycerol phosphate synthase/phosphoribosylanthranilate isomerase TrpCF | 313.49 | 1014.74 | 1.69 |
| BW25113_RS07300 | 1,2-phenylacetyl-CoA epoxidase PaaD | 4.15 | 13.04 | 1.63 |
| BW25113_RS06570 | Tryptophan synthase subunit alpha TrpA | 980.72 | 2830.09 | 1.53 |
| BW25113_RS19600 | Ketol-acid reductoisomerase IlvC | 1048.11 | 191.42 | -2.45 |
| BW25113_RS17915 | Branched chain amino acid ABC transporter substrate-binding protein LivK | 368.22 | 73.05 | -2.33 |
| BW25113_RS11255 | Lysine-specific permease LysP | 330.04 | 89.57 | -1.88 |
| BW25113_RS11115 | ABC transporter permease proline/glycine betaine transport system OpuBB | 30.60 | 9.56 | -1.67 |
| BW25113_RS20875 | Lysine-sensitive aspartokinase 3 LysC | 917.26 | 295.92 | -1.63 |
| BW25113_RS02505 | Glutaminase 1 GlsA | 36.85 | 11.86 | -1.63 |
| BW25113_RS09210 | Glutamate dehydrogenase GdhA | 2041.84 | 720.94 | -1.50 |
| **^a^***E. coli* cultures were grown in triplicate in M9-glucose medium under DEA613.  **^b^**cut-off 1: log_2_fold change ≤ 1.5 or ≥ 1.5  **^c^**cut-off 2: q-value ≤ 0.05 | | | | |

| **Table S11.** Differentially-expressed genes related to glyoxals and aldehydes metabolism in the DEA613-adapted strain T56-1 versus wt both grown under DEA613.**^a,b,c^** | | | | |
| --- | --- | --- | --- | --- |
|  | | **FPKM average** | |  |
| **Locus** | **Annotation** | **wt** | **T56-1** | **Log_2_ fold change** |
| BW25113_RS07420 | Aldehyde dehydrogenase AldA | 61.28 | 497.14 | 3.02 |
| BW25113_RS15585 | Glyceraldehyde 3-phosphate reductase YghZ | 54.62 | 261.20 | 2.26 |
| BW25113_RS18630 | Aldehyde dehydrogenase AldB | 15.16 | 69.63 | 2.19 |
| BW25113_RS14560 | Lactaldehyde reductase FucO | 15.04 | 65.01 | 2.10 |
| **^a^***E. coli* cultures were grown in triplicate in M9-glucose medium under DEA613.  **^b^**cut-off 1: log_2_ fold change ≤ 1.5 or ≥ 1.5  **^c^**cut-off 2: q-value ≤ 0.05 | | | | |
